# Promising images of love: a qualitative-ethnographic study about the mediatised memories of weddings

**DOI:** 10.12688/openreseurope.16521.1

**Published:** 2023-11-16

**Authors:** Marie-Therese Mäder

**Affiliations:** 1 Dipartimento di studi umanistici, Universita degli Studi di Macerata, Macerata, Marche, 62100, Italy

**Keywords:** Weddings, rite of passage, mediatisation, spaces of memory, gender, video interviews, photo elicitation

## Abstract

**Background:**

Digital media play a central role in contemporary weddings, be it during the preparation, the ritual itself or afterwards. Specific moments of the wedding day are made memorable through photographs and videos. But how do couples manage their wedding photos and videos and what role do these media play in awakening memories of religious and secular dimensions of the event? These questions are developed in the theoretical frame of mediatisation and memory processes. This paper argues that the mediatisation of weddings influences how a wedding is memorized and results in a partial standardization of memories of the event, in which neither cultural nor religious differences nor sexual orientation play a crucial role.

**Methods:**

This interdisciplinary qualitative-ethnographic research is based on twenty-seven semi-structured video recorded interviews conducted in Italy, Germany, and Switzerland with homo- and heterosexual married couples from different cultural-religious backgrounds. The couples shared their wedding album or video and developed a wedding narrative by looking at the photographs and videos.

**Results:**

Taking photos during the wedding becomes an important part of the ritual, independent of any cultural or religious background, gender, and sexual orientation. The ceremony itself is emphasized in the photos and the video. This homogenisation of the rite of passage equally occurs in the photographic representations of weddings and in turn influences how the wedding is remembered. The findings further reveal that heterosexual marriage representations reinforce gender stereotypes. The brides/wives of heterosexual couples are significantly more invested in the production and reception of wedding media, whereas homosexual couples participated in the conversation equally.

**Conclusions:**

The homogenization of representation and memory becomes part of the rite of passage’s collective memory that connects the individual couples. The mediatisation of weddings strengthens the feeling of belonging to a community that transcends cultural-religious identities.

## Plain language summary

Digital and electronic media play a central role in contemporary weddings, be it during the preparation, the ritual itself or afterwards. Specific moments of “the most important day of one’s life” are made memorable through photographs and videos. But how do couples manage their wedding photos and videos and what role do these media play in awakening memories of religious and secular dimensions of the event? I argue that wedding photos and videos influence how a wedding is memorized and results in a partial standardization of memories of the event, in which neither cultural nor religious differences nor sexual orientation play a crucial role.

## Introduction

Digital and electronic media play a central role in contemporary weddings, be it during the preparation, the ritual itself or afterwards. By means of photographs and videos the bridal couples and hired media professionals (or photographically gifted friends) capture moments of “the most important day” in each couple’s lives. By doing so, specific moments are made memorable through the media produced around the wedding in order to shape the memories of the bridal couple and their guests in the future. The current paper is focused on the following questions: how do couples manage their wedding photos and videos? What role do these media play in awakening memories of religious and secular dimensions of the event? This paper argues that the mediatisation of weddings influences how a wedding is memorized and results in a partial standardization of memories of the event, in which neither cultural nor religious differences play a crucial role. In contrast differences could be observed regarding gender and sexual orientation and how the participants engaged in the conversations. The female participants of heterosexual couples were more familiar with the photos and often took more speaking time. Whereas homosexual couples participated in the conversation more balanced and often also rather emotional compared to heterosexual couples. ^1^

The meaning and function of wedding photos and videos, like any other private media, essentially differ depending on whether you were part of the event or not. ^2^ For a fuller understanding of these media you need to ask the people who participated in the event. Therefore, this research is based on twenty-seven semi-structured interviews that are between 50 and 90 minutes long with couples from different cultural-religious backgrounds: Christian, Jewish, Muslim as well as interfaith and non-religious couples in Italy (11 interviews), Germany (7 interviews), and Switzerland (9 interviews). The countries have been chosen because their marriage laws and also the discourses around marriage differ. In Germany, same-sex marriage has been legal since 2017 and civil union since 2001. ^3^ In Switzerland, same-sex marriage was accepted in a referendum in 2021 by 64.1% of the voters with a turnout of 52.6% and came into effect legally from 1 ^st^ July, 2022. ^4^ Italy differs from many other European countries in the legalisation of same-sex marriage. Legal union of same-sex couples was first introduced in 2016, but this does not have the same legal status as marriage because adoption and artificial insemination is prohibited by law for couples in such unions. ^5^

The first paper of this paper will provide an overview of the relevant literature presented, that focuses on how values and norms are expressed in representations such as images and texts as well as how wedding practices participate in political discourses. Then the theoretical frame is laid out in which mediatisation, memory processes, and the spaces of memory are considered. The analysis results of the of the video-recorded conversations is discussed in the second part of the paper. During the conversations the couples showed their wedding album or video and developed their wedding narrative by looking at the photographs and videos. In the conclusion, the results of the analysis are synthesized in the context of memory and mediatisation.

### Literature on weddings

The research question scrutinizes weddings from a contemporary perspective with a focus on the media that are produced during the wedding. These wedding media include photos and videos that are produced professionally, semi-professionally, or non-professionally. About this specific topic, no scholarly literature has been published so far. However, there are quite a few relevant publications that show diverse aspects of wedding representations, in a historic or contemporary perspective across the globe. (1) The first group of publications reflects cultural-political dimensions in a historical perspective by means of case studies identifying how wedding norms are created. ^6^ Three anthologies focus on different historical aspects that show how weddings and the institution of marriage are closely related, how power, marriage, and eroticism interact and have always been instrumental for political agendas. ^7^ These historical accounts of marriage and wedding rites enable us to understand how marriage and the wedding ceremony are connected with symbols of power and how they transform over time. Additionally, they highlight that historically, weddings have scarcely been related to love and romance but more often to improve or stabilize the politics of the privileged.

(2) The literature about weddings from a contemporary perspective mostly considers problematic areas based on the politics of same sex marriage, globalisation, and capitalism. Hilde Schäffler’s rich ethnographic study about the wedding business in Austria shows how weddings are commercialised in wedding fairs, by wedding planners and by wedding day directors. ^8^ Sabine Exner Krikorian reconstructs in her monograph how the traditional heterosexual constellation of weddings is transformed in political discourse. ^9^ Another volume also deals with same sex marriage and how it was accepted in the United States. ^10^ While a second anthology questions the institution of marriage in general. ^11^ The authors discuss further relationship possibilities than the classical monogamous style, notably polygamy and polyamory. These contemporary accounts of weddings and marriage bring to light that on the one hand the wedding industry is mainly focused on monogamous and heterosexual relationships. On the other hand, this heteronormative and western concept of weddings is currently challenged in culture and politics. ^12^

(3) The last group of publications discuss wedding representation in the media such as wedding shows, films, or wedding magazines. There are quantitative studies, qualitative studies, and analytical approaches. Females tend to consume more romantic media content than male viewers. ^13^ But in the case of dating shows, as a quantitative psychological study shows, some men watch them to learn how to date. ^14^ One study asks why people watch wedding reality shows (WRTV) and how it endorses romantic beliefs. Entertainment is the main reason, but also the romantic endorsement of “love conquers all”. Further literature about weddings and media focus on wedding representations. The monograph *White Weddings: Romancing Heterosexuality in Popular Culture* ^15^ by Chrys Ingraham scrutinizes how the white wedding is applied to confirm and maintain heterosexuality as the dominant norm of relationships in U.S. society. Specifically, the depiction of weddings in popular media perpetuates and idealizes the concept of traditional heterosexuality, while simultaneously reinforcing rigid gender roles and societal expectations. In 2008, when Ingraham’s revised monograph was published same sex marriage hadn’t been legalized in any of the countries featured in this current study (Italy, Germany, Switzerland). Therefore, it needs to be verified if it is still the case, that heteronormativity “is the view that institutionalized heterosexuality constitutes the standard for legitimate and expected social and sexual relations.” ^16^

“Wedding Reality TV Bites Black: Subordinating Ethnic Weddings in the South African Black Culture” ^17^ and “Portrayal of Religion in Reality TV Programming: Hegemony and the Contemporary American Wedding” deal with dominant representations. ^18^ The first paper applies a multimodal discourse analytical study with a focus on professional production and representation of weddings of Black people in WRTV in South Africa: “Whether by accident or intentionally, predominantly *Our Perfect Weddings* and *Top Billing Weddings* feature Black women whose idea of beauty seems to be rooted in whiteness specifically regarding hair.” ^19^ This fact, Lindani Mbunyuza-Memani considers, is symbolic violence in the wedding media, because WRTV represents Black looks consistently as inferior. The norm of stereotyped Christian weddings is scrutinized in Erika Engstrom’s paper about *A Wedding Story* (TLC, US 1996–2005). ^20^ The content analysis of 85 episodes and 42.5 hours of recorded footage reveals a Christian dominance. This reinforces the idea that a stereotyped Christian marriage is the norm.

Finally, with a focus on the representation of weddings in the media the authors and editors of the special issue of the *Journal for Religion, Film and Media* offer the following definition of the interaction between weddings and the media: “The public is a collective space and tradition is based on collective norms, and weddings also sit between these categories: they are semi-private, semi-traditional, and semi-individual. Wedding rituals thus form a point of intersection at which basic elements of living together coincide.” ^21^ This definition of wedding rituals combined with Gerard van Gennep’s definition of weddings as *rites de passage ^22^* are the starting point for this study of the mediatisation of weddings and how couples engage with their wedding photos and videos.

The literature about weddings in the media discussed above mainly considers the dominant representations of weddings. These prevailing characteristics of weddings are Christian, heterosexual, white, and western. From this it can be concluded that weddings are depicted in the media as highly normative, reinforcing heteronormativity which leads to the homogenization and the suppression of diversity. For the current study the question arises if (semi-)private wedding media are different from entertainment media and more diverse? This question leads us to the theoretical frame of the study that considers the concepts of mediatisation and memory.

### Mediatisation of weddings

When looking at memory processes in the context of wedding media, the concept of “mediatisation” is central. It is concerned with the way in which media change human interactions and experiences. ^23^ With regard to wedding practices, the mediatisation of the wedding event captures and communicates particular gender, social, cultural, religious, and economic values of individuals and groups that are later remembered and affirmed when looking at the images of the event. These communicative practices by means of the media are not only relevant after the wedding event but play a central role even during the wedding celebrations. Specifically, taking pictures functions as an important activity during the wedding. The couples spend extra time, sometimes hours, with the photographer at pre-selected places. The cell phones of the guests and the professional equipment of the photographer have become a fixed component of wedding essentials, alongside the bridal flower-bouquet and the cake. ^24^ The wedding guests form groups to be photographed and ask the couple to kiss in order to catch the moment with their cameras. Afterwards, the wedding guests often send their photos to the couple, perhaps in the form of a photo album or the photos are uploaded to a platform to make them accessible to all. Likewise, the couple sends pictures of the event to their guests in the form of thank-you cards.

All these interactions mentioned above communicate messages by means of images that constitute the concept of mediatisation. People exchange meanings that in a cultural studies perspective are expressed in practices. ^25^ In the current study these practices are part of the *rite of passage* of a wedding. Making images during the wedding influences not only how the wedding is experienced in the present. But it also shapes how it is reconstructed in the future when someone looks at the photos and videos alone or together with family members and guests. Mediatisation encapsulates the whole process of analysing how media are used to structure, communicate, and remember an event like a wedding, as well as how the experiences of the participants are influenced and changed. ^26^ In view of the fact that remembering the wedding through looking at photos and videos are at the core of the current research question it needs to be considered further how these processes may be understood.

### Memory processes and memory spaces of wedding media

From the perspective of mediatisation, remembering the wedding by looking at pictures and videos not only includes the present moment of looking at the pictures but also remembrance of the moment in the past when they were taken, as Hans Belting states:

The symbolic act of perception in front of a photograph consists in an exchange of gazes. We recall the gaze, which is in turn recalled in the photograph. In this sense, photography is a medium between two gazes, and a part of this mediality consists in the time that lies between the recorded and the recognizing gaze. ^27^

Combining these two gazes, the gaze of the photographer that perpetuates the photo and the gaze of the receivers who are looking at the image transforms the moment when the photo has been taken:

We see the world with the gaze of another, a past gaze, but we trust that it could also be our present gaze. The same world always looks different when it is seen at a different time. We look upon the world in an image that represents the same gaze with which we, too, experience the world. The difference is that it has now achieved permanence. ^28^

Looking at wedding photos results in a double assurance, namely the permanence of the new status that is expressed in the ritual and the confirmation in the photos that have achieved permanence, according to Belting. To remember the wedding by looking at the photos is a practice that is experienced by the person who is looking at the images. The practice is shared with the other guests that remember the day, and by people that couldn’t be present but are told about the wedding. These different memory processes play a central role in understanding the function of wedding media.

With respect to the function of wedding media in the memorization process, the concept of memory is captured as two-dimensional: on the one hand there is the individual memory of the ritual with the unique experience of this day. On the other hand, there is the cultural memory of the wedding, whereby photos and videos play a supporting role to keep the memory alive for the couple and its wedding guests. These two dimensions of memory merge into each other. They shape the couples shared identities but also their personal identities regarding gender, cultural, religious, social, and economic boundaries. ^29^

By remembering through photos and videos individuals and groups need to do something with them. Therefore, in distinction from the informal memory that lacks cultural characterstics, “[c]ultural memory, by contrast, always depends on a specialized practice, a kind of ‘cultivation’.” ^30^ This understanding of the two-dimensional concept of memory, cultural and individual, and specifically the notion of a “specialized practice” highlights that memory needs to be performed in practices that feed the memory to keep it alive and present. Because memory is connected to practices one can consequently speak of “doing-memory”. Jay Winter uses the term “performative acts” to describe such practices of remembrance that are performed and expressed in emotions:

“The performance of memory is a set of acts, some embodied in speech, others in movement and gestures, others in art, others still in bodily form. The performative act rehearses and recharges the emotion, which gave the initial memory or story imbedded in it its sticking power, its resistance to erasure or oblivion. Hence affect is always inscribed in performative acts in general and in the performance of memory in particular.” ^31^

According to Winter, memory is a set of bodily acts like speech, movement and gestures that connects the memory with emotions. These emotions provide sticking power in order that we don’t forget. Memory processes consist of performative acts that are mediated through the body. In this theoretical frame, the function of wedding photos and videos is to trigger the memories and emotions. However, memory behaves similar to mediatisation processes as meaning making practices. Not only that, but the photos themselves are important tools to remember the wedding day. In many cases the couples also remember how the photos were shot. Often, they describe the making-of the photos of the bridal couple as a particularly emotional moment.

Each couple participates in a shared cultural memory through their wedding photos. The reception of their photos and the memory of the day and the stories of their making contribute to the cultural practice of the wedding ritual. The ritual as well as its media production, distribution, and reception practices become part of a shared cultural identity.

As it has been laid out thus far, taking and looking at photographs of a wedding take part in different memory processes, namely individual and cultural memory, respectively. Both are performed in different moments that in the current approach are systematized as *spaces of memories.* Wedding photos are taken and the videos are filmed during the wedding. Sometimes even pre-wedding shoots take place. The production of wedding media can be understood as one memory space. Another space is constituted by the photos and videos themselves as the memory space of representation. They often follow or play with certain aesthetic norms of wedding depictions. Photos are presented sometimes in photo albums, as a pack of unsorted photos, or in a digital folder on a hard drive or memory stick. As already mentioned, these images and videos are often also shared with friends and families, online or in person, in the third memory space of distribution. The fourth and last space is the space of reception in which the photos and videos are looked at by different people; the couple themselves, the wedding guests or the people that couldn’t be present at the wedding. In this case the couple shares their memory with other people with the help of the photos and videos.

These spaces of memory namely the production, representation, distribution, and reception include certain practices which also overlap. The concept of spaces of memory is derived and further developed from the concept of communication spaces. The latter is embedded in a semio-pragmatic approach that connects the meaning making process of representations with its contexts of production, distribution, and reception. ^32^ In other words: the meaning of wedding photos and videos is developed through their context of how they are produced, distributed, and received. The following analysis is segmented in these four spaces of memory that are emotionally charged. It considers how memory is performed and how it achieves its “sticking power”.

## Methods

The current study is based on conversations in the four languages German, Italian, English, and Swiss-German. Twenty heterosexual and seven homosexual couples couples were interviewed in Germany, Italy, and Switzerland between April 2022 and February 2023. Ten couples married in a civil ceremony only, six of whom are same-sex couples. Only one gay couple married in a traditional religious ceremony. The sample includes seventeen traditional religious and ten alternative ceremonies that took place between 1968 and 2022. Among the 54 participants there are 24 participants female and 30 are male by self-report regarding sex. ^33^ The aim was to meet married couples in three different countries, of diverse cultural and religious backgrounds, different sexual orientations, and different years of marriage, in order to achieve a diversity of political, cultural, and religious contexts of social actors. This diversity of contexts sharpens the study’s focus on the media and how people remember the event. The procedure allows us to understand the commonalities and differences of memory and reception processes.

The conversations were recorded with a camera applying a method that is rooted in visual ethnography. ^34^ The conversations have been video recorded with an Iphone 13 max, a tripod, and an external microphone that has been attached to the Iphone. Most of the meetings took place in the participant’s homes or apartments. The couple sat next to each other looking at the wedding photos. The setting was as such that the couple as well the photos could be recorded in the same frame. During the conversations he social actors were observed by the camera as they look at and comment on their wedding photos and videos. This method refers to photo-elicitation of which a variety of applications are available. ^35^ In the current research project “reflexive photography” was applied, meaning, couples were asked to tell their wedding story by showing and commenting on their photos. There was some variability in the structure of the conversations because the weddings differ. Some couples got married in a civil service whilst other weddings took place in a religious ritual. In Italy, for example, civil and religious marriage can be done in the church where the priest gives the sacrament and also acts as registrar. This is not possible in either Switzerland or Germany.

The conversations were structured into three parts: the first part was about the preparation, the second about the wedding itself, and the third about the meaning of their wedding media. The couples were asked, for example, about the role of their wedding photos and videos in remembering their wedding. Examples of questions asked include (for the full surveys, see *Extended data* ( Mäder, 2023w)): On what grounds did they chose the photographer? Which is their favourite photo of their wedding day, and why? What was the most important moment? What would you change in retrospect if you could? When did they last look at the photos, and if there are particular occasions when they have looked at them. However, for the majority of the conversations, a question-and-answer format wasn’t applied but rather the couple recounted their own wedding narrative.

Methodologically, the camera as a means of collecting data was crucial. During the subsequent analysis that has been conducted with the software atlas.ti (version 23.2.0) the recordings allow the viewer to see which photo the couple was talking about. ^36^ Additionally, the emotional reaction to the images are not only expressed by words but also by the couple’s body-language. Finally, there are many moments in which nobody is talking but there are still reactions to the photos and interactions between the couple which are then caught by the camera. The video protocols are transcribed verbatim ^37^ and evaluated by combining grounded theory and sociological hermeneutics of knowledge. ^38^ The analysis focused on the way the couple comments upon the photos and videos of their wedding and how they construct their wedding narrative with the help of these media sources, and if specific patterns related to gender could be observed during the conversations. Additionally, the representation strategies of the wedding photos and videos are analysed and compared.

## Results

The three memory spaces of production, representation, and reception structure the following analysis of the conversations with the couples. The space of distribution is included in the reception space because the way in which the photos and videos are accessible and distributed overlap. Looking at the photos or videos requires that they had been distributed. Additionally making wedding media available is based on a modality of distribution. In the space of production the focus is on how the couples remember the photographer and the taking of pictures during the wedding. In the space of representation, the photographs and videos are analysed scrutinizing wedding photography standards. Finally, in the reception space, the couple’s reactions and comments on the photos and videos are summarized. In all four memory spaces the “sticking power” of memory performance is elaborated and achieved through emotions. Therefore, the analysis of the conversations focuses on the emotional experiences in the production, representation, reception, and distribution experience of wedding media.

### Memory space of production

Ten couples didn’t hire a photographer for the wedding whilst seventeen hired a photographer or a professional photographer was among their friends and family. But even in cases where there was no professional photographer, the photographing and posing for pictures still played an important role. During the conversations there were two different perceptions on the photographic process during the wedding. There were moments in which the photographer wasn’t noticed. This was especially during the ceremony when many couples didn’t realize the presence of the photographer. In some cases the photographer provided instructions before the ceremony that the couple should slow down or even freeze for a moment in order to be able to “catch the important moment”. This was for example the case during the signing of the wedding contract or the exchange of rings as one husband explained:

Man: The only moment I really paid attention to him was there. And also there. Because he told me, before you sign, just slow down, just wait with the pen. Because many do it far too quickly and then it’s gone.
Woman: And then you don’t have a photo of it at all.
Man: He said the same thing [like during the signing] to me while putting on the ring. "Pause for a round when you’re there. And try not to cover it with your hand, but try it nicely. Then there will be a good photo of it too." I tried to think of these things. But otherwise I didn’t notice him taking any photos. ^39^

This example shows how the taking of pictures can become a part of the ceremony by influencing its procedure. The couple naturally and without questioning included the photographer’s instructions for the sake of having a photograph of this important moment. The ceremony itself is being altered through this. But the couple play the game by pretending that freezing is part of the ceremony. They perform for the photographer to receive in return the pictures as a memory of this central moment of the ceremony.

There are other important moments that most want to be captured photographically. There is the kiss after the ceremony. Usually, there is no photo of a kissing couple before the ceremony. The photo of the kissing newlywed couple exists in almost every wedding photo collection as if the kiss after the wedding has a different quality to the ones that took place before the wedding. Another very important moment that is photographed in most weddings is the exit from the ceremony venue. These photographs are dynamic by nature. In the wedding video of two women who conducted their civil union in Rome in 2016 at the registry in Campidoglio reveals how willing the couples are to follow the instructions of the photographers. ^40^ The couple actually didn’t hire a professional photographer but friends took the photos and gave the wedding album to the couple afterwards as a gift. Almost at the end of the wedding video the couple exits the civil registry office and the guests are clapping. Suddenly the couple turns around and reenters the building to exit again. There is a voice who recalls them “Piano, piano!” meaning they should exit slowly, to carry out the role better the second time. One wife explains: “They had told us we had left too fast and hadn’t taken any pictures.” And the other wife adds: “I have to say that we laughed a lot.” ^41^ The video includes this double exit as a funny moment to be remembered. During the conversation both women were whole heartedly laughing about this memory that obviously achieved sticking power.

It was striking that the couples often told me stories about the making of the pictures. They remembered the shooting as a special and exciting moment with some almost miraculous coincidences. The wedding shoot of a German couple who married in 2011 close to Frankfurt/M took place after a thunder storm. ^42^ The professional photographer returned in the evening to take some pictures of the couple, the best man and the maid of honor. The couple remembers the shoot vividly and they enthusiastically told me how it went:

Woman: And at seven o’clock the photographer came and then asked: ''Should I come again tomorrow? Do you want to get dressed again tomorrow or do we want to take pictures afterwards?" And then we said: "We’re going to take pictures now. We’ll get rubber boots." [Laughter]
Man: Yes, the rubber boots, we weren’t the only ones who ruined their shoes – I think – the whole wedding party did.
[…]
Woman: It’s like, yes, well, that’s a meadow that was particularly great because it wasn’t mowed, but it’s a place like that, I really like being there because you can see so far and we have also said: ''We don’t want to go back to the castle somehow [where we married] to take photos'', but somehow a place that – maybe for me – has a meaning and you thought it was good too? ^43^

It is remarkable that the couple agreed on all the details, the atmosphere, and how they emotionally felt about the photo shoot even though the wedding took place twelve years ago. When looking at the series of portraits on this meadow and telling me about the thunderstorm and the rubber boots that they wear, their enthusiasm was obvious. They laughed a lot about their story although the wife was more engaged. The husband showed his approval with a guarded smile. The photo with the rubber boots had been sent as a Thank-you card to the guests and also the grandmother chose that one instead of the serious one with the wedding shoes. Here the photo shoot became an important moment to be remembered and perpetuated in the “rubber boots photo”. By its reception and the couple’s distinct memory of the photographer’s first gaze, the picture achieved what Belting describes “permanence”. ^44^ The photographer’s gaze through the lens of the camera is inscribed in the photo. When the couple is looking at the picture the image achieves a permanent quality beyond this distinct moment of the act of photography. Additionally the act of remembering the emotions during the photo shoot provides “sticking power” for the couple.

*Memory space of representation*

The memory space of representation includes the actual photos and videos that have been taken. There are different ways in which the photos are presented. Some couples owned an elaborate wedding album with additional drawings and ornaments. One couple just brought their wedding photos in envelopes and apologized that the photos haven’t been pasted into an album. There were seven couples in all that didn’t have a photo album. Only four couples actually had a professionally produced video, one of them wasn’t pleased about the result, so they didn’t want to show it. Three other couples owned a video that was shot by friends or family members. The couples with a video married at different times and countries. One of the couples with the professional video had additionally saved some images on their cell phones that had been posted in social media channels. Three couples only stored their photos digitally and also apologized for doing so. In the conversations the wedding album as a standard to present the photos has been positively evaluated. If there is not a wedding album, the couple often apologizes because digital images are perceived as less valuable. According to a wedding photographer, digital photos often get lost. This is why he always strongly recommends couples produce a wedding album that doesn’t get lost. ^45^

In all cases of the heterosexual couples, the wife was more invested in the production of the wedding album. In the following section, some cases are highlighted that show the importance of presenting and appreciating the photos appropriately. A young Italian couple, just recently married in 2022, showed me their “provisional” wedding album. The wife compiled the album with the pictures that their friends sent her during their honeymoon. She didn’t want to wait half a year for the photographer’s official and professional wedding album. ^46^ The woman collected their friends’ cellphone photos on an online platform and arranged them in an online tool that provides software to compile albums. The albums can be ordered as print outs. She looked at their friends’ photos during the whole honeymoon and on their way back, she compiled the album.

Another German couple who married in 2022, received a selection of almost 500 photos from the photographer. During the conversation, the woman took the lead in telling the narrative of the wedding. Often, I had to address the husband explicitly in order to know how he perceived a moment that has been depicted in the photograph. In the middle of our meeting, in response to the question of whether a specific photo represents the situation as he remembers it, he affirms and adds: “Yes, I watch them way too seldom. I may only [have looked at] them [the photos] for the third time now, but [my wife] I think she looks at them almost every day, it feels like that.” ^47^ Both are laughing about this comment, the woman feels a bit caught. But also the husband is embarrassed about the fact that his wife knows the photos much better than him although she denies that she looks at them “every day”.

Another younger couple showed me their album that contained a lot of texts. These texts had been transcribed by the wife from the two videos. But the album begins with the story of their engagement that is written by the husband. ^48^ The photo album is extremely elaborate and it is obvious that the woman invested a lot of time in compiling the photos and transcribing the texts. The couple married in three ceremonies. The first was at the registry, the second in a member’s garden house who blessed them in a self-designed wedding ceremony that was “like in a film as my wife wanted” as the husband commented. ^49^ As the couple are members of the Church of Jesus Christ of the Latter-day Saints the third ceremony took place in the temple in Zollikofen in Switzerland. Photography and filming is strictly forbidden in the temple. So, the “filmlike” garden ceremony could be seen as a kind of proxy ceremony to be photographed to replace the temple ceremony.

In general, it can be stated that the ceremony itself is very densely depicted compared to other parts of the wedding. The style of the reception doesn’t matter, there are equally as many photos of an alternative ceremony as of a traditional religious or a civil ceremony. There is one couple who married in an alternative ceremony that took place in the wine cellar of a wedding venue. The place is reminiscent of a crypt. The wedding started at 5pm and lasted until midnight. The ceremony with a master of ceremony who was the husband’s cousin took about 45 minutes. From their 478 photos selected by the photographer 97 depict the ceremony, specifically the ring ritual that according to the husband refers to a Turkish engagement ritual. The husband is half Turkish and his wife is Swiss but they live together in Germany. Also, for this wedding the wife admitted that she felt much more in charge than the husband. ^50^

Often when the couples are asked to choose one of their favourite pictures, one is from the wedding ceremony. Even the couple who celebrated the wedding in the Catholic Church in a “ *rito disgiunto*”, which means according to the couple that one person receives the marriage sacrament and the other doesn’t. Both agreed that they liked the photos in the church during the ceremony the most.

An Italian couple who showed me their professionally produced wedding video commented a lot while watching the video. ^51^ But they stopped talking when the Catholic ceremony in the church with the priest was played. The wedding video is about 21 minutes long and the ceremony is shown for 8 minutes which is in a blatant temporal disproportion to the rest of the wedding celebrations. Taking into account that the wedding started at 11 am and ended close to midnight, the ceremony of less than an hour was only a small temporal part of that day. In the reception, the party with the guests, becomes less important and the official ceremony occupies a prominent place. Furthermore, in this case, the wedding ceremony has been highlighted in the wedding video and influences also how and which moments of the wedding are remembered. The same could be observed in the conversations. Because there were so many pictures of the ceremony, the conversation dominantly revolved around this topic.

The depiction of the ceremony is also highlighted in the civil registry office as in the case of the lesbian couple who married in Rome in Campidoglio. ^52^ They received an offer from a videographer who filmed the ceremony to buy the video afterwards which they accepted. The video highlights the same moments as in traditional religious or alternative marriage: it shows the speech of the master of ceremony, then the vows of the couple, the exchange of rings, the signing of the contract, the kiss after the wedding ceremony, and the exit from the civil registry office. And finally, there is a kind of a “victory photo” after the ceremony in almost all cases independent of what kind of ceremony it has been. The “victory photo” shows the couple usually holding hands and uplifting the other’s arm as we know it from sports when the winner celebrates his or her achievement. The photo is often very dynamic and cheerful so that the couples usually like it a lot and choose it as their favourite photo.

### Memory space of reception

The space of reception in which the couples look at the photos and videos, takes a special role in the research process. First, it is reproduced during the interviews and second, the two perspectives of the couples are actively synchronized during the conversation. Often there was an idea that there were different perceptions of the day, but these perceptions could not be in opposition to each other. Therefore the couples tried to synchronize their memories. Further, the couples, specifically the ones who married in a traditional religious ceremony, wanted to explain in detail the rite and highlighted its significance. This was the case with the Swiss couple who married in Mumbai in a Hindu ceremony, ^53^ the liberal orthodox Swiss couple in the synagogue in Basel, ^54^ the Italian couple with the Greek-orthodox ceremony in Venetia, ^55^ and two more Italian couples who married in the Catholic church in a traditional rite. ^56^ The husband of one of them explained that they belong to the conservative strand “Communione e Liberazione” (CL) of the Catholic church and that is the reason the ceremony was specifically significant:

It was a great thing for everyone. How exceptional the ceremony was because the ceremony was truly significant. Six witnesses, three mine and three her’s. Six friends I wanted to have. My sister who read. Another five priests who celebrated. Yes because we experience that – it is strong catholic.
[…] So you understand that they [the guests] were all a little surprised when they attended this very religious but significant ceremony. ^57^

They were equally proud of their Catholic heritage and the symbolic dimension of the wedding which the photographer sought to depict in the style of the photos. The husband reflects on the quality of the photos:

But then it was nice because if you see the photos, you’ll see that the photos, which are the same thing that strikes those from what you’ll see, is that he made us this beautiful catalog where, however, we are not always in close-up, not with our faces. But there is the context, the friends, the church. Sure point he photographs a part of the Church. So the catalog isn’t just the bride and groom who are part of a community, of a structure and then at a certain point they also bring us. It also brings us back – too much am I telling? ^58^

It was striking that the husbands of these couples who married traditionally were especially invested, not only in explaining the ritual wedding procedure in detail, but also in clarifying their wives’ statements or sometimes even correcting them if they thought that she didn’t explain something precisely enough.

Another recurrent topic in the reception process were discussions about how frequently the photos and videos had been viewed. Many looked at their photos with friends and family members after the wedding to remember the wedding day. Many couples share the pictures with their guests. One couple even told me that they have displayed pictures of other weddings but none of their own. There was only one couple who told me that they hadn’t looked at their enormously large wedding album for 27 years. And it bothered them that their daughters didn’t want to look at it. But they were impassioned to show their wedding album for the interview that was extremely elaborate with many oversized photos and an emphasis on the ceremony. ^59^

Mostly there is one person who is more enthusiastic in looking at the pictures. Specifically in the case of the heterosexual couples, it is the woman who talks more about the photos as the example of the German couple shows that has been discussed above. ^60^ The husbands often explained the meaning of the symbolic practices that had been depicted in more detail.

Whilst watching the videos and looking at the photos with the couples, some couples explained that these photos look romantic or meaningful. But they also had a lot of fun during their celebrations like this Italian wife remembers:

I remember my wedding, I had so much fun, I always danced, always, I never stopped. And I remember it really funny. The video on the other hand gives me more of a romantic feeling, like there’s – all these romantic scenes, right? With this cheesy music and so on. So maybe the video made me-- I mean it makes me relive the romance part, but I remember it a lot more [laughter], a lot funnier. ^61^

Additionally, the couples mentioned that the photos and video allowed them to see and remember things they didn’t perceive during the day because they were so busy and distracted.

One last observation is that almost all the gay couples expressed sadness and responded emotionally to the photos or the video. Some of them even cried. Their experience of being excluded from this ritual left emotional traces that came to the surface during the conversations. In general during most conversations the watching of the album together was an emotionally intense and personal experience. The couples often appreciated the opportunity to tell their wedding story and sent thanks after the interviews.

## Conclusion: the wedding as a picture story and photo romance

This study about the mediatisation and the memory of weddings considered the role of wedding media such as photos and videos in remembering the *rite de passage*. The twenty-seven conversations with couples from Germany, Italy and Switzerland brought to light that wedding photos and videos are highly standardized representations. The memory of their production and reception experiences shows surprising similarities between the couples independent of the couple’s age, year and country of wedding, sexual orientation, or religious-cultural background. In the following section, the most significant results from different spaces of memory are reviewed. The conclusion highlights four aspects. First, the memory space of representation that is expressed in the emotional style of the photos. Second, the observations about gender are brought together. Third, the memory space of production is considered as part of the rite of passage. Fourth, and finally, some critical thoughts about the study are formulated and further questions are raised.

(1)   As was shown in the study, memory processes are practices that achieve “sticking power” through emotions. These emotions are provoked in different spaces. The production of photos is often experienced as a special event with the photographer. There are many photos in a “romantic style” that highlight a tender and close connection between the couple in a warm light. These depictions aim to depict and highlight an emotional quality of the moment. Also many photos are taken during the ceremony, be it traditionally religious, an alternative rite, or a civil wedding. The multitude of photos or the extensive footage of the ceremony highlights this part of the wedding in the couple’s memories. Even in the conversation with the couples the ceremony has often been extensively discussed. Finally, it is notable that the couples who only owned digital photos of their wedding apologized for this. While couples with elaborate albums were proud to show this “piece of art”.

(2)   Regarding gender and wedding media mostly, in heterosexual couples, the wives were more invested, be it in compiling the photo albums, looking at them, or talking about the wedding. During these conversations, the husbands often had to be explicitly addressed, in order to find out more about their opinion and their experiences during the wedding. Although in the case of traditional religious weddings the husbands often took the lead in explaining how the religious ceremony is structured. They talked about the symbolic meaning of the different practices, readings, blessings, or props. In cases in which the wife explained the ceremony, the husbands often added details or even corrected her which was seldom the case the other way round. The gay couples tended to share their talking time more equally and took turns in telling their wedding narrative. It seemed that gay couples are equally invested in the pictures, and the images of the wedding itself and the reception, was something very emotional and of high importance in a different way than in the case of heterosexual couples. While the heterosexual couples often expressed that their wedding was a bit “different” but when looking at the photos it seemed quite traditional. The gay couple’s wedding was different because they belonged to the first generation who were able to legally marry. The political dimension is of an important value in the rite of passage. Often gay couples playfully applied traditional wedding themes and adapted it to their lifestyle. They ordered gay cake figures online or their dress was in most cases the same or at least in a way corresponding to each other. The emotionality during telling their wedding narrative was often overwhelming.

(3)   In the memory space of production, the importance of taking the pictures was striking and how much time many couples invested or at least took some time alone with the photographer. The wedding photo session becomes a part of the *rite of passage* that is captured in the different photo subjects. They show the kissing couple, the couple in the sunset, in a fancy car, or after the ceremony in front of the registrar’s office, the church doors, or wherever the ceremony took place. Specifically, the ceremony is densely photographed in standardized images like the exchange of the rings, signing of the wedding contract, or the “victory photo” of the couple after the wedding. Not all of these photo moments are specifically emotional but in their reception an emotional value and importance is highlighted, because they have been depicted. Photos of the ceremony have often been chosen by the couples as one of their favorites. It shows that the depiction itself reinforces a moment’s positive value and provides it’s sticking power. The photographic style and quantity of specific moments influences in what way the *rite de passage* is remembered. Additionally in the memory space of reception, as one couple explicitly mentioned, the wedding media allow the couples to see and experience moments of the wedding day they missed out. The couples often mentioned that they felt a lot of tension and the day went by very quickly. In this case, the wedding media facilitate the couple to not only remember but also experience the ceremony or the party from an insider observer perspective. In this way, media enlarge the wedding experience and in the words of Belting “achieve permanence”. They transcend the *rite of passage* to transform it into a consistent value that in the reception is experienced as confirming and self-assuring. The media themselves reinforce the couple’s wedding promises and their reception allows viewers to repeat and revive the emotional quality. Additionally, because wedding images are highly standardized, the memory of the event is homogenized. This event could be observed in the individual conversations. The couple created the wedding narrative together and adapted their different experiences to each other in order to create one memory. It is remarkable how many similarities between the couples’ memories could be observed independent of religious, cultural, age, and educational background as already mentioned. This homogenization becomes part of a collective memory of the rite of passage that connects the individuals. Additionally, it strengthens the feeling of belonging to a community that transcends the cultural-religious identity.

The study focused on married couples from different cultural backgrounds, gender, and sexual orientations. The two main commonalities between the couples were the photos or videos of their wedding. And secondly, they are still married. Having a conversation with separated couples would have been another way of looking at the wedding media and their meaning. One separated woman told me that the only thing she will do with her wedding photos is “to burn them”. To include separated couples and also widowers could provide further insights into the role of media in remembering the rite of passage of weddings.

## Ethics and consent

Ethical approval was granted by the Ethics Committee of the university of Macerata on 22 February 2022 and by H2020-MSCA-IF_2020 (D1.1–D1.5), on 4th July 2022. Written informed consent for publication of the participants details was obtained from the participants.

## Data Availability

Due to protection of privacy and the sensitivity of interview footage, the data can only be shared based on the explicit consent of the participants. The data includes wedding videos and photos in which religious affiliation, sexual orientation and personal details are discussed. It is stated in the written consent form, that the data will not be made open unless the participants agree. The transcriptions of the interviews can be accessed by submitting a request to the author via email, which should detail for what purposes the transcriptions would used. This project contains the following underlying data: Zenodo: Video Interview with transcript, married couple, Italy https://doi.org/10.5281/zenodo.8248549 ( Mäder, 2023a) Zenodo: Video interview with transcript, married couple, Italy https://doi.org/10.5281/zenodo.8248660 ( Mäder, 2023b) Zenodo: Video interview with transcript, married couple, Italy https://doi.org/10.5281/zenodo.8248820 ( Mäder, 2023c) Zenodo: Video Interview with Transcript, married Couple, Switzerland https://doi.org/10.5281/zenodo.8181259 ( Mäder, 2023d) Zenodo: Video interview with transcript, married couple, Germany https://doi.org/10.5281/zenodo.8186799 ( Mäder, 2023e) Zenodo: Video interview, married couple, Switzerland https://doi.org/10.5281/zenodo.8249679 ( Mäder, 2023f) Zenodo: Video interview with transcript, married couple, Switzerland https://doi.org/10.5281/zenodo.8248393 ( Mäder, 2023g) Zenodo: Video interview, married couple, Italy https://doi.org/10.5281/zenodo.8190190 ( Mäder, 2023h) Zenodo: Video interview with transcript, married couple, Switzerland https://doi.org/10.5281/zenodo.8247614 ( Mäder, 2023i) Zenodo: Video interview with transcript, married couple, Italy https://zenodo.org/record/8178945 ( Mäder, 2023j) Zenodo: Video interview with transcript, married couple, Switzerland https://doi.org/10.5281/zenodo.8248443 ( Mäder, 2023k) Zenodo: Video interview with transcript, married couple, Germany https://doi.org/10.5281/zenodo.8247717 ( Mäder, 2023l) Zenodo: Video interview with transcript, married couple, Italy https://doi.org/10.5281/zenodo.8190308 ( Mäder, 2023m) Zenodo: Video interview with transcript, married couple, Germany https://doi.org/10.5281/zenodo.8181413 ( Mäder, 2023n) Zenodo: Video Interview with Transcript, Married Couple, Germany https://doi.org/10.5281/zenodo.8190269 ( Mäder, 2023o) Zenodo: Video interview, married couple, Switzerland https://doi.org/10.5281/zenodo.8249632 ( Mäder, 2023p) Zenodo: Video interview with transcript, married couple, Germany https://doi.org/10.5281/zenodo.8247577 ( Mäder, 2023q) Zenodo: Transcript of video interview, married couple, Italy https://doi.org/10.5281/zenodo.8190245 ( Mäder, 2023r) Zenodo: Video interview with transcript, married couple, Italy https://doi.org/10.5281/zenodo.8249246 ( Mäder, 2023s) Zenodo: Video interview with transcript, married couple, Italy https://doi.org/10.5281/zenodo.8246842 ( Mäder, 2023t) Zenodo: Video Interview with transcript, married couple, Switzerland https://doi.org/10.5281/zenodo.8247782 ( Mäder, 2023u) Zenodo: Video interview with transcript, married couple, Germany https://doi.org/10.5281/zenodo.8184184 ( Mäder, 2023v) Zenodo: Questionnaires_English_German_Italian https://doi.org/10.5281/zenodo.8249440 ( Mäder, 2023w) This project contains the following extended data: Couples_English_questionnaire.pdf Couples_German_questionnaire.pdf Couples_Italian_questionnaire.pdf Data are available under the terms of the Creative Commons Zero "No rights reserved" data waiver (CC0 1.0 Public domain dedication).

## Footnotes

^1^ The current study adheres to the SAGER guidelines regarding sex and gender in research. See Shirin Heidari *et al.*, “Sex and Gender Equity in Research: Rationale for the SAGER Guidelines and Recommended Use,” *Research Integrity and Peer Review* 1, no. 1 (May 3, 2016): 5, https://doi.org/10.1186/s41073-016-0007-6. Sex is understood as a biological classification that has been assigned based on self-report of the participants in the study. Gender is understood as a socially constructed category. It defines social processes forming the body through clothing, behaviour, visual representation, sexual orientations a.o. See Anna-Katharina Höpflinger, Anne Lavanchy, and Janine Dahinden, “Introduction: Linking Gender and Religion,” *Women’s Studies: An Interdisciplinary Journal*, 2012, 619/620.

^2^ Roger Odin, “The Home Movie and Space of Communication,” in *Amateur Film Making: The Home Movie, the Archive, and the Web*, ed. Laura Rascaroli, Gwenda Young, and Barry Monahan (Bloomsbury Publishing USA, 2014), 15–26.

^3^ Sabine Exner-Krikorian, *Recht Auf Liebe: : Eine Diskursanalyse über Die Gleichgeschlechtliche Ehe in Deutschland.* (Wiesbaden: Springer Fachmedien Wiesbaden GmbH, 2022), 23–30.

^4^ Bundeskanzlei BK, “Politische Rechte,” accessed June 26, 2023, https://www.bk.admin.ch/bk/de/home/politische-rechte/pore-referenzseite.html.

^5^ Alessia Donà, “Somewhere over the Rainbow: Italy and the Regulation of Same-Sex Unions,” *Modern Italy* 26, no. 3 (August 2021): 261, https://doi.org/10.1017/mit.2021.28.

^6^ Angelika-Benedicta Hirsch, *Warum die Frau den Hut aufhatte: kleine Kulturgeschichte des Hochzeitsrituals* (Göttingen: Vandenhoeck & Ruprecht, 2008); John Witte, *The Western Case for Monogamy over Polygamy*, Cambridge Studies in Law and Christianity (New York, NY: Cambridge University Press, 2015).

^7^ Reinhard Lebe, *Ein Königreich als Mitgift: Heiratspolitik in der Geschichte* (München: DtTaschenbuch-Verlag, 2000); Line Cecilie Engh, *The Symbolism of Marriage in Early Christianity and the Latin Middle Ages: Images, Impact, Cognition* (Amsterdam: Amsterdam University Press, 2019), https://doi.org/10.1017/9789048537150; Julia Moses, *Marriage, Law and Modernity: Global Histories* (London: Bloomsbury Academic, 2018); Johannes Gießauf, Andrea Penz, and Peter Wiesflecker, *Im Bett mit der Macht: Kulturgeschichtliche Blicke in die Schlafzimmer der Herrschenden* (Köln: Böhlau Verlag, 2011), https://doi.org/10.7767/boehlau.9783205791133.

^8^ Hilde Schäffler, *Ritual als Dienstleistung: die Praxis professioneller Hochzeitsplanung* (Berlin: Reimer, 2012).

^9^ Exner-Krikorian, *Recht Auf Liebe*.

^10^ Darel H. Paul, *From Tolerance to Equality: How Elites Brought America to Same-Sex Marriage* (Waco Texas: Baylor University Press, 2018).

^11^ Ronald C. Den Otter, *Beyond Same-Sex Marriage: Perspectives on Marital Possibilities* (Lanham: Lexington Books, 2016).

^12^ Lucy Williams, *Global Marriage: Cross-Border Marriage Migration in Global Context*, Migration, Minorities & Citizenship (Basingstoke: Palgrave Macmillan, 2010).

^13^ Marie-Therese Mäder, “»Ich wollte Schwestern, nicht nur einen Mann«: Zum Verhältnis von Gender und Religion in Reality Shows,” in *Religion und Gender* (transcript Verlag, 2022), 93–95, https://doi.org/10.1515/9783839457733-004.

^14^ Veronica Hefner, “Tuning into Fantasy: Motivations to View Wedding Television and Associated Romantic Beliefs,” *Psychology of Popular Media Culture* 5, no. 4 (October 2016): 307–23, https://doi.org/10.1037/ppm0000079.

^15^ Chrys Ingraham, *White Weddings: Romancing Heterosexuality in Popular Culture*, 2nd ed. (New York: Routledge, 2008).

^16^ Ingraham, 44.

^17^ Lindani Mbunyuza-Memani, “Wedding Reality TV Bites Black: Subordinating Ethnic Weddings in the South African Black Culture,” *Journal of Communication Inquiry* 42, no. 1 (January 1, 2018): 26–47, https://doi.org/10.1177/0196859917726047.

^18^ Erika Engstrom and Beth Semic, “Portrayal of Religion in Reality TV Programming: Hegemony and the Contemporary American Wedding,” *Journal of Media and Religion* 2, no. 3 (August 1, 2003): 145–63, https://doi.org/10.1207/S15328415JMR0203_02.

^19^ Mbunyuza-Memani, “Wedding Reality TV Bites Black,” 42.

^20^ Engstrom and Semic, “Portrayal of Religion in Reality TV Programming.”

^21^ Anna-Katharina Höpflinger and Marie-Therese Mäder, “‘What God Has Joined...’ Editorial,” *Journal for Religion, Film and Media (JRFM)* 4, no. 2 (November 3, 2018): 16.

^22^ Arnold Van Gennep, *The Rites of Passage*, 2nd ed. (Chicago: The University of Chicago Press, 2019), 116–45.

^23^ Marie-Therese Mäder, *Mormon Lifestyles: Communicating Religion and Ethics in Documentary Media* (Nomos, 2020), 44–46; Friedrich Krotz, “Mediatisierung,” in *Handbuch Cultural Studies und Medienanalyse*, ed. Andreas Hepp et al. (Wiesbaden: Springer Fachmedien, 2015), 439–452, https://doi.org/10.1007/978-3-531-19021-1_45; Stig Hjarvard and Mia Lövheim, eds., *Mediatization and Religion: Nordic Perspectives* (Göteborg: Nordicom, 2012), 21–44.

^24^ Krotz, “Mediatisierung,” 443.

^25^ Stuart Hall, “The Work of Representation,” in *Representation: Cultural Representation and Signifying Practices*, ed. Stuart Hall, Jessica Evans, and Sean Nixon (Los Angeles, CA: SAGE, 2013), 1–47.

^26^ „Generally speaking, mediatization is a concept used to analyze critically the interrelation between changes in media and communications on the one hand, and changes in culture and society on the other.” See Nick Couldry and Andreas Hepp, “Conceptualizing Mediatization: Contexts, Traditions, Arguments,” *Communication Theory* 23, no. 3 (2013): 6, https://doi.org/10.1111/comt.12019.

^27^ Hans Belting, *An Anthropology of Images: Picture, Medium, Body* (Princeton, New Jersey: Princeton University Press, 2011), 154, https://doi.org/10.1515/9781400839780.

^28^ Belting, 154.

^29^ Jan Assmann and John Czaplicka, “Collective Memory and Cultural Identity,” *New German Critique*, no. 65 (1995): 125–33, https://doi.org/10.2307/488538.

^30^ Assmann and Czaplicka, 131.

^31^ Jay Winter, “The Performance of the Past: Memory, History, Identity,” in *Performing the Past: Memory, History, and Identity in Modern Europe*, ed. Karin Tilmans, Jay Winter, and Frank van Vree (Amsterdam: Amsterdam University Press, 2010), 12.

^32^ Mäder, *Mormon Lifestyles*, 39–41; Roger Odin, *Les Espaces de communication: Introduction à la sémio-pragmatique* (Grenoble: Presses universitaires de Grenoble, 2011), 15.

^33^ The difference between male and female participants is based on the willingness to participate in the study of lesbian and gay couples. Gay couples were more likely to participate. In the current study all the participants’ sex is based on self-report as already mentioned before. The partcipants’ gender identity is congruent with the self-reported sex.

^34^ Sarah Pink, *Doing Visual Ethnography*, 4th ed. (London: SAGE Publications, 2021), 81–170.

^35^ Gillian Rose, *Visual Methodologies: An Introduction to Researching with Visual Materials*, 3rd ed. (London: Sage, 2012), 304–316; Francesco Lapenta, “Some Theoretical Thoughts and Methodological Views on Photo-Elicitation,” in *The SAGE Handbook of Visual Research Methods* (London: SAGE Publications, 2011), 202–11, .

^36^ The quotes from the conversations with the couples are copied from atlas.ti. The corresponding footnotes are generated by atlas.ti and refers to the transcript that has been saved to zenodo. A free alternative software to atlas.ti is CATMA see “CATMA,” accessed September 25, 2023, https://catma.de/.

^37^ Elizabeth J. Halcomb and Patricia M. Davidson, “Is Verbatim Transcription of Interview Data Always Necessary?,” *Applied Nursing Research* 19, no. 1 (2006): 40, https://doi.org/10.1016/j.apnr.2005.06.001.

^38^ Ronald Kurt and Regine Herbrik, “Sozialwissenschaftliche Hermeneutik und hermeneutische Wissenssoziologie,” in *Handbuch Methoden der empirischen Sozialforschung*, ed. Nina Baur and Jörg Blasius (Wiesbaden: Springer Verlag, 2014), 473–91; Andreas Boehm, “Grounded Theory - wie aus Texten Modelle und Theorien gemacht werden,” ed. Andreas Boehm, Andreas Mengel, and Thomas Muhr (Konstanz: UVK Univ.-Verl. Konstanz, 1994), 121–40; Jörg Strübing, “Grounded Theory und Theoretical Sampling,” in *Handbuch Methoden der empirischen Sozialforschung*, ed. Nina Baur and Jörg Blasius (Wiesbaden: Springer Verlag, 2014), 457–72.

^39^ 20:2 ¶ 67 – 69 in VIW_22_CH_010223_III_ts.docx. The couple married in 2022 in Switzerland. The groom was 25 years and the bride 24 years old. The quotes from the video recorded interviews are numbered and abbreviated as following: VIW_22_CH_010223 (video interview no. 22, the six digit number represents day, month, and year of recording).

^40^ The couple was 56 and 37 years old at their wedding (see video interview VIW_26_IT_110223).

^41^ 149:3 ¶ 73 – 77 in VIW_26_IT_110223_IV_ts.docx. The conversation has been conducted in Italian and translated in English by the author (see video interview VIW_26_IT_110223).

^42^ The groom was 34 years and the bride 28 years (see video interview VIW_07_DE_160923).

^43^ 39:3 ¶ 86 – 112 in VIW_07_DE_160923_IV_ts.docx. The conversation took place in German and translated in English by the author (see video interview VIW_07_DE_160923).

^44^ Belting, *An Anthropology of Images*, 157.

^45^ The conversation with the wedding photographer took place in Naples/IT in March, 2022 and has been recorded.

^46^ 154:1 ¶ 44 in VIW_25_110223_V_ts.docx. The bride was 30 years and the groom 32 years old. The conversation has been conducted in Italian and translated in English by the author (see video interview VIW_25_IT_110223).

^47^ 127:1 ¶ 116 – 118 in VIW_15_DE_311022_VIII_ts.docx. The groom was 35 years and the bride 32 years old. The conversation has been conducted in German and translated in English by the author (see video interview VIW_15_DE_311022).

^48^ 20:10 ¶ 9 – 12 in VIW_22_CH_010223_III_ts.docx (see video interview VIW_22_CH_010223).

^49^ 18:6 ¶ 65 – 67 in VIW_22_CH_010223_II_ts.docx (see video interview VIW_22_CH_010223).

^50^ See video interview VIW_15_DE_311022.

^51^ The groom was 40 years and the bride 32 years old at their wedding that took place in Italy in 2022 (see video interview VIW_11_IT_290922).

^52^ See video interview VIW_26_IT_110223.

^53^ The groom and bride were both 22 when they married in 1988 (VIW_21).

^54^ See video interview VIW_04_CH_260522.

^55^ See video interview VIW_24_IT_110223.

^56^ See video interviews VIW_11_IT_290922 and VIW_02_IT_070422 (see the following footnote).

^57^ 58:13 ¶ 142 – 146 in VIW_02_IT_070422_I.docx (see video interview VIW_02_IT_070422).

^58^ 58:14 ¶ 186 in VIW_02_IT_070422_I.docx (VIW_02_IT_070422).

^59^ See video interview VIW_02_IT_070422.

^60^ See video interview VIW_15_DE_311022.

^61^ 62:4 ¶ 75 in VIW_11_IT_290922_V_ts.docx (see video interview VIW_11_IT_290922).
