# Promising images of love: a qualitative-ethnographic study about the mediatised memories of weddings

**DOI:** 10.12688/openreseurope.16521.2

**Published:** 2024-12-05

**Authors:** Marie-Therese Mäder

**Affiliations:** 1 Dipartimento di studi umanistici, Universita degli Studi di Macerata, Macerata, Marche, 62100, Italy

**Keywords:** Weddings, rite of passage, mediatisation, spaces of memory, gender, video interviews, photo elicitation

## Abstract

**Background:**

Digital and electronic media play a central role in weddings, capturing everything from the preparation to the ceremony itself and beyond. These media make specific moments of the wedding day memorable. But how do couples manage their wedding photos and videos, and what role do these media play in evoking memories of the event's religious and secular dimensions? These questions are explored within the theoretical framework of mediatisation and memory processes. This paper argues that the mediatization of weddings evokes emotions that strengthen the impact of these memories. Media shape how these events are recalled, leading to a partial homogenization of memories, where cultural and religious differences, as well as sexual orientation, become less prominent.

**Methods:**

This interdisciplinary qualitative-ethnographic research is based on twenty-seven semi-structured video recorded interviews conducted in Italy, Germany, and Switzerland with homo- and heterosexual married couples from different cultural-religious backgrounds. The couples shared their wedding album or video and developed their wedding narrative by looking at the photographs and videos.

**Results:**

Taking photos during the wedding becomes an important part of the ritual, independent of any cultural or religious background, gender and sexual orientation. The ceremony itself is emphasized in the photos and the videos. This homogenisation of the rite of passage equally occurs in the photographic representations of weddings and in turn influences how the wedding is remembered. The findings further confirm that heterosexual marriage representations reinforce gender stereotypes. The brides/wives of heterosexual couples are significantly more invested in the production and reception of wedding media, whereas homosexual couples participated in the conversation rather equally.

**Conclusions:**

The homogenization of representation and memory becomes part of the rite of passage’s cultural-collective memory that connects the individual couples. The mediatisation of weddings strengthens the feeling of belonging to a community that transcends cultural-religious identities.

## Plain language summary

Digital and electronic media play a central role in contemporary weddings, be it during the preparation, the ritual itself or afterwards. Specific moments of “the most important day of one’s life” are made memorable through photographs and videos and the emotions they evoke. But how do couples manage their wedding photos and videos and what role do these media play in awakening memories of religious and secular dimensions of the event? I argue that wedding photos and videos influence how a wedding is memorized and results in a partial standardization of memories of the event, in which neither cultural nor religious differences nor sexual orientation play a crucial role.

## Introduction

Digital and electronic media play a central role in contemporary weddings, be it during the preparation, the ritual itself or afterwards. By means of photographs and videos the bridal couples hire media professionals (or photographically gifted friends) to capture moments of “the most important day” in each couple’s lives. Weddings take place between the private and the public sphere. “The public is a collective space and tradition is based on collective norms, and weddings also sit between these categories: they are semi-private, semi-traditional, and semi-individual. Wedding rituals thus form a point of intersection at which basic elements of living together coincide.” ^1^ This definition of wedding rituals combined with Gerard van Gennep’s definition of weddings as *rites de passage ^2^* are the starting point for this study of the mediatisation of weddings and how couples engage with their wedding photos and videos. Wedding photos and videos make specific moments memorable and at the same time shape the memories of the bridal couple and their guests in the future.

Therefore, the current paper is focused on the following questions: How do couples manage their wedding photos and videos? What role do these media play in awakening memories of religious and secular dimensions of the event? Are there differences between the recollection of religious and secular weddings through wedding photos and videos? This paper argues that the mediatisation of weddings influences how a wedding is memorized and results in a partial homogenisation of memories of the event, in which neither cultural nor religious differences play a crucial role. Even the memories of weddings from different eras show striking similarities. By contrast, differences could be observed regarding gender and sexual orientation and how the participants engaged in the conversations. The female participants of heterosexual couples were more familiar with the photos and often took more speaking time. Whereas homosexual couples participated in the conversation more equally and often more emotionally, when compared with the heterosexual couples in the study. ^3^

The meaning and function of wedding photos and videos, like any other private media, essentially differ depending on whether you were part of the event or not. ^4^ For a fuller understanding of these media you need to ask the people who participated in the event. Therefore, this research is based on twenty-seven semi-structured interviews that are between 50 and 90 minutes long with couples from different cultural-religious backgrounds: Christian, Jewish, Muslim as well as interfaith and non-religious couples in Italy (11 interviews), Germany (7 interviews), and Switzerland (9 interviews). The countries have been chosen because their marriage laws and also the discourses around marriage differ. In Germany, same-sex marriage has been legal since 2017 and civil union since 2001. ^5^ In Switzerland, same-sex marriage was accepted in a referendum in 2021 by 64.1% of the voters with a turnout of 52.6% and came into effect legally from 1st July 2022. ^6^ Italy differs from many other European countries in the legalisation of same-sex marriage. Legal union of same-sex couples was first introduced in 2016, but this does not have the same legal status as marriage because adoption and artificial insemination is prohibited by law for couples in such unions. ^7^

The first part of this paper will provide an overview of the relevant literature presented that focuses on the relation between production, representation, and reception of wedding photography and how they shape the memories of the event. Then the theoretical frame is laid out in which mediatisation, memory processes, and the spaces of memory are considered. These concepts establish a framework within which the analysis of the video-recorded conversations is discussed in the second part of the paper. During the conversations the couples showed their wedding album or video and developed their wedding narrative by looking at the photographs and videos. In the conclusion, the results of the analysis are synthesized in the context of memory and mediatisation.

### Literature on wedding media

The research question scrutinizes weddings from a contemporary perspective with a focus on the media that are produced during the wedding. These wedding media include professional, semi-professional, or non-professional photos and videos. The existing literature about wedding photography deals with (1) norms and values, (2) memories, (3) the ritual dimension, and (4) emotions.

(1) The scholarly literature on the topic considers how wedding photo- and videography (re-)produces, confirms, transforms, and challenges norms and values such as gender, heterosexuality, and consumerism. ^8^ Several studies critically analyze and deconstruct these norms and values by applying a variety of methods and sources. Michele M. Strano investigates the role of wedding photography as a ritual performance that functions as a significant tool for both reinforcing and challenging social norms, and expectations. ^9^ Not all wedding photos challenge the values of marriage, gender, and consumerism. According to Lili Corbus Bezner the wedding album actually “affirms the institutions of marriage and family, reaffirming such values in subsequent viewings.” ^10^ The photographically captured rituals are a humanistic need to reach beyond the everyday banal world into the sacred. Bezner’s rather affirmative understanding of wedding media contrasts with more critical approaches. In an autoethnographic study Marc A. Ouellette deconstructs the materiality of the wedding and the norms of love and intimacy. The vanishing point of the study leads to a six hour photo session. The author and his wife previously bought all the necessary items for dressing up as a wedding couple in second hand shops. In his understanding wedding photos serve as promotional tools for the event and the proof that the day happened. ^11^

The representational norms of wedding pictures can also serve as a means to adapt to a cultural context. Nhi T. Lieu identifies representational norms in wedding photos of Asian-American wedding couples that align with a US American lifestyle. Lieu considers these photos as threatening to the US American culture instead of becoming part of it. ^12^ In my qualitative ethnographic approach, I distinguish between religious and secular values and norms inscribed in photos of the ceremony location. I observe that the differences between values and representational norms associated with civil and religious spaces are often blurred. The photographic representations of the venues and how the couples evaluate them are highly normative and characterized by an enchantment of not only religious but also secular spaces. ^13^

To summarize wedding photography not only produces and confirms but may also question societal and cultural norms and values.

(2) Norms and values of wedding photography influence the couples’ and their guests’ memories of the event as several studies consider. With the technological evolution, photography gained new possibilities to create memories. According to Charles Lewis “[w]edding photographs are powerful because they are traditional, professional, personal, and seemingly accurate renditions of reality as they help couples remember a key period in their social and personal lives.” ^14^ Specifically, the possibility of taking photos on location instead of in the studio transformed wedding photography. This candid photography provided the photographers with a defining role in the ceremony and the whole event. They arrange the motifs and the couple’s poses. The couple follow the instructions of the wedding photographer both because they trust in the specialist’s directions, and also because they expect beautiful photos of their wedding day as a result. According to Lewis, wedding photography not only elevates the status of the event but it also shapes how it is remembered.

Photographs, because they are permanent representations that can influence one’s memory of the ceremony, tend to increase the significance of the wedding event. However, the process of photography also alters perceptions of the event. It imposes itself on the actual ceremonial event, and its conventionalized images may tend to mystify memories of the actual rites of the ceremony. In other words, the photographs do not necessarily represent the actual rites and emotions that were involved but how the entire ritual should have been [performed], […]. ^15^

Through the engagement of the photographer the ritual itself becomes altered. The photographer develops a conventionalized narrative and interprets the wedding according to the dominant social and cultural context, such as adding emotions with specific techniques of double exposure. ^16^ Lewis additionally considers the production of wedding photos as interpreting the world. They are powerful tools to confirm “social realities as class dominance and gender inequality” that are expressed in the consumption process of specific goods, such as bridal gowns, tuxedos, cakes, flower bouquets, and hired limousines. These goods are part of the ceremony and are depicted in a conventionalized photographic style that enchants the wedding in the memory process. ^17^ Cele C. Otner and Elizabeth H. Pleck even consider the production of memories during the wedding as being almost as important as the ritual itself. “For all of these reasons, the memories that the wedding produces are almost as important as the magic generated by the occasion.” ^18^ Photos are often the most effective and emotionally valuable souvenirs of the day as their display on the walls and in frames on the living room’s side board proves. Wedding photos not only confirm family ties, they also “provide the bride and groom with tangible evidence that they had their day to shine as the stars of their social network and provide them with a means of reviving their belief in ‘happily ever after.’” ^19^

(3) Otnes and Pleck further describe the function of these idealized representations as souvenirs of a ritual that connects the past with the present to remember good times in difficult times. ^20^ Likewise, the romantic lavish wedding provides a “repository of memories of this magic and romance, and offers the promise of perfect (e.g. boundless and guilt free) consumption.” ^21^ In this case photos have a double function. They legitimate luxurious consumption because it is part of the ritual transformation captured forever in photographs. At the same time, photos store memories of the magical and romantic rite of passage.

These stored memories are presented in wedding albums that function to teach viewers about the correct behaviour during a wedding and what the ritual should look like. “Sharing the album, then, becomes a powerful mnemonic tool as the ritual is reenacted, stories added, encouraging cultural continuity, community, and the teaching of certain matrimonial behaviours.” ^22^ Wedding albums allow one to learn and remember what a wedding ritual should look like and to take part in handing down a tradition. Through wedding albums people also remember how the wedding ritual is appropriately celebrated.

According to Bezner, the photographer co-designs the rite in a “shining language”. By doing so she or he takes part in the ritual practice that is passed down not only to future bridal couples but also to the next generation. Many wedding albums are structured in three stages that resonate with Victor Turner’s concept of the rite of passage following Arnold van Gennep. ^23^ The wedding album begins with separation. The middle phase is the transitional period, and finally the couple is reincorporated into the community at the end. Therefore the photos and the presentation of the ritual in albums function as a social and cultural memory.

Strano identifies an additional connection between the wedding ritual and the photos. She conceives weddings as a ritual performance that consequently ritualizes the memory of the rite of passage “through the use of photography, becoming more formalized, repetitive, symbolic, and performative.” ^24^ The author considers photos in a similar way to Otnes and Pleck as being “the most powerful and prevalent communication mediums used to ensure that others ‘will remember’ through the ‘tangibility of things’.” ^25^ According to Strano the ritualizing of memory through the photographic technology enables “formalized depictions of the past that perpetuate social groups and values” ^26^. The individual may choose to participate in this group ritual by reproducing standard wedding motifs like the newly wed picture of the kissing couple after the ceremony.

At the same time the couple can question the norm of the kissing motif, for example, by exaggerating the pose. Strano’s study illuminatingly explores both how couples negotiate symbolic norms of wedding photos during the production process, as well as showing how wedding photos may likewise comply with these norms for an audience. ^27^ Therefore, the production of wedding photos serves two distinct purposes. First, it captures the couple and their guests in a visual record of the ritual. Second, it visually communicates the event to a specific audience, namely the couple, their families and friends and those who were unable to attend the celebrations. In both cases the photographs are intended to serve as a tangible memento of the ritual.

The process of looking at the photos and sharing the memories is accompanied by the development of a narrative of that day, which in turn is influenced by the style of the photographic motifs. “Photographs in general and personal photographs in particular do not appear as self-sufficient memory content but as fragments in dire need of contextualization. So they initiate active memory work instead of replacing it.” ^28^ Jens Ruchatz understands remembering as an active process. The images serve as stimuli that are complemented with information from the individuals’ own memory in order to activate their recollection of the event. The chronological sequence of the three phases of the wedding rite allows the viewer to retrospectively reconstruct the event in greater detail, depending upon the extent to which the photo album complies with this structure. Likewise, the various moments of the wedding may be more readily integrated into the appropriate sequence. Therefore, the ritual structure of the photos supports the social memory of the wedding by structuring the individual memories similarly. Even if the wedding is understood as a personally designed event, it makes the photos readable and ritualizes the memory process. ^29^

(4) Additionally, emotions support the memory process as James Walsh and Matthew Wade show in their analysis of 132 wedding videos. In the case of wedding videos, sound is an effective means to steer the stickiness of emotions that “constitute a powerful aural framing device that aids personal and collective memory construction.” ^30^ Again the personal is distinguished from the collective memory, consistent with Ruchatz’s analysis of the difference between individual and collective memory. ^31^ This difference also proves to be crucial in the current study. The couple shares a collective memory of the day and at the same time they are individuals who may each have different experiences and emotions. How far these divergent experiences are synchronized or are accepted as such tells us something about the context of the photos, the active process of remembering, and how much individuality is possible. “In its rejection of visual conflict, wedding photography may even have redemptive power to bring the hope of cohesion and stability to social groups.” ^32^ Both in photographing and in looking at the images, this cohesion and redemptive power may be possible. Emotions are another effect mentioned in relation to memory processes, as will be discussed later. The photos use different stylistic means to control emotions compared with a video. The captured emotions of the guests and the bride and groom, such as tears of joy, are important. Romantic shots of the couple in soft light or at sunset are also popular emotional motifs.

The discussion of previous studies about wedding photos and videos can be summarised as follows. The studies primarily took place in the U.S., Canada, and Australia. They focused on the production process, with the photographer at the center who influenced the ritual and the representation of wedding photo motifs that reflect certain norms and values. Additionally, they consider how wedding media influence the ritual structure and the memories of the bridal couple and the guests. In these memory processes emotions play a crucial role. After all, wedding media are produced for an audience that should be satisfied with the photos and, in the best case, be enthusiastic about the images.

The production, representation, and reception of wedding media shape how weddings are remembered. In the current study these processes are theoretically framed by the concept of mediatisation, which offers valuable insights into the ways mediatised communication works and how it transforms cultural practices like weddings. The field study asked how couples revisit their memories, both individual and collective, while viewing images of their wedding day. Therefore, the concept of mediatisiation is enlarged with memory processes. The following section will develop the concepts of mediatisation and memory, both individual and cultural memory, and explore how they interact in the context of wedding media.

### Mediatisation and mediation of weddings

Mediatisation is concerned with the way in which media change human interactions and experiences in a long-term perspective. ^33^ With regard to wedding practices, the mediatisation of the wedding event captures and communicates particular gender, social, cultural, religious, and economic values of individuals and groups that are later remembered, affirmed or rejected, when looking at the images of the event. These communicative practices by means of the media are not only relevant after the wedding event but play a central role even during the wedding celebrations. Specifically, photography is an important activity during the wedding, with the photographer playing a central role. According to Lewis “(t)he photographic story of the wedding day did not develop individually with each new wedding event; the story was mostly created by the photographer, not necessarily experienced by the couple.” ^34^ As the current study shows the couples are more than willing to spend extra time, sometimes hours, with the photographer. They visit pre-selected places to be photographed in pre-defined motifs.

The cell phones of the guests and the professional equipment of the photographer have become a fixed component of wedding essentials, alongside the bridal flower-bouquet and the cake. ^35^ The cake and the bouquet will not retain their freshness for long after the day is over, but the photos will become the most significant relics of the event. ^36^ The wedding guests form groups to be photographed and ask the couple to kiss in order to catch the moment with their cameras. Afterwards, the wedding guests often send their photos to the couple, perhaps in the form of a photo album or the photos are uploaded to a platform to make them accessible to all. Likewise, the couple sends pictures of the event to their guests in the form of Thank-you cards.

All these practises mentioned above communicate messages by means of images. These communicative-symbolic interactions during and after weddings are summarised under the term “mediation”. According to Mark Deuze the media form and transform meanings and “social and cultural forces operate freely according to various logics, with no predictable outcome. The process of mediation inevitably influences or changes the meaning received, […]”. ^37^ To put it in a nutshell, mediation refers to the ongoing practice of symbolic communication, “its passing through technologically-based infrastructures of transmission and distribution (‘media’). By contrast, ‘mediatization’ refers more specifically to the role of particular media in emergent processes of socio-cultural change.” ^38^

In the current study these conscious and carefully coordinated mediation practices are part of the rite of passage of the wedding. Making images during the wedding influences not only how the wedding is experienced in the present. But it also shapes how it is reconstructed in the future when looking at the photos and videos alone or together with family members and guests.

Eventually the production and reception of wedding photos fundamentally changes the cultural practice of the rite of passage which is all subsumed under the term mediatisation. “Generally speaking, mediatization is a concept used to analyse critically the interrelation between changes in media and communications on the one hand, and changes in culture and society on the other.” ^39^ The change takes place on the micro level of the individual that turns into groups on a meso level when several people or even whole communities communicate through media collectively. ^40^ And finally, the macro level provides the big picture of the change through media in society.

Transferred to weddings, the three levels of socio-cultural change can be summarised as follows. The micro and the meso level of mediatisiation concern the weddings as they are organized by the couple and family in a partly individual and partly collective endeavour. Also, the wedding photographer per se is an individual who is part of a larger group of specialists. The ways in which individuals and groups interact with the media vary, but when it comes to weddings, they adapt to stylistic norms as previously shown. The macro level asks then how wedding media change the socio-cultural meaning of weddings.

The relation between the communicative-symbolic interaction with photos, *mediation* and the socio-cultural change, *mediatisation*, plays a central role in the memory processes of weddings. In the context of mediation, the stylistically normed wedding photography illustrates the way events are shaped by the media. At the level of mediatisation, questions regarding the distinctions and similarities between the photographs and memories, the role of the cultural and religious context of the depictions, and the transformation of such contexts through wedding media over time are significant. ^41^ The current study argues that the cultural practice of adhering to photographic stylistic conventions level out the cultural-religious differences between the institutionalised ceremonies over time on a macro level. ^42^ Therefore both approaches, a cultural (social constructionist) and an institutionalist, to mediatisation are relevant whereas the processuality of both is crucial. ^43^ Both encapsulate the whole process of how media are used to structure, communicate, and remember an event.

### The mediated memory of weddings

As already pointed out remembering the wedding by looking at pictures and videos often not only includes the depicted moment but also the remembrance of taking the photos. In many cases the couples describe the making-of the photos of themselves, as a bridal couple, as a particularly emotional moment. Looking at wedding photos results in a double assurance, namely the permanence of the new status that is expressed in the ritual and the confirmation of that status in the photos that, according to Hans Belting, have achieved permanence. ^44^ To remember the wedding by looking at the photos is a practice that is experienced by the person who is looking at the images. The practice is shared with the other guests that remember the day, and by people that couldn’t be present but are told about the wedding. These different memory processes highlight the fundamental role of wedding media in memory processes.

With respect to the function of wedding media in the memory process, the concept of memory is captured as two-dimensional: on the one hand there is the individual memory of the ritual with the unique experience of this day. On the other hand, there is the cultural memory of the wedding, whereby photos and videos play a supporting role to keep the memory alive for the couple, their wedding guests, and future generations. ^45^ These two dimensions of memory merge into each other. They shape the couple’s shared identities but also their personal identities regarding gender, cultural, religious, social, and economic boundaries. ^46^

By remembering through photos and videos individuals and groups need to do something with them. Therefore, distinct from informal memory that lacks cultural characteristics, “[c]ultural memory, by contrast, always depends on a specialized practice, a kind of ‘cultivation’.” ^47^ This understanding of the two-dimensional concept of memory, cultural and individual, and specifically the notion of a “specialized practice” highlights that memory needs to be performed in practices that feed the memory to keep it alive and present. Because memory is connected to practices one can consequently speak of “doing-memory”. Jay Winter uses the term “performative acts” to describe such practices of remembrance that are performed and expressed in emotions:

The performance of memory is a set of acts, some embodied in speech, others in movement and gestures, others in art, others still in bodily form. The performative act rehearses and recharges the emotion, which gave the initial memory or story imbedded in it its sticking power, its resistance to erasure or oblivion. Hence affect is always inscribed in performative acts in general and in the performance of memory in particular. ^48^

According to Winter the performative acts of memory processes are mediated through the body. By means of bodily acts like speech, movement and gestures the memory relates to emotions. These emotions provide sticking power in order that we don’t forget. ^49^ In this theoretical frame, the function of wedding media is to trigger emotions in order to remember. ^50^ However, memory and mediation complement each other as meaning making practices. The photos structure the memory process and recall the photo sessions during the wedding. By doing so each couple participates in a shared cultural memory through their wedding photos that are conspicuously normed. The control over these memory processes, therefore, is “a balancing act in which photo-images ‘enculturate’ personal identity.” ^51^ The reception of the photos, the individual and cultural memory of the day, and the stories of their making contribute to the meaning making practice of the wedding ritual in which memory and identity intersect. The ritual as well as its media production, distribution, and reception practices not only become part of a shared cultural memory but also a shared identity.

Individual and cultural memory are performed in different moments that in the current approach are systematized as *spaces of memories*. Pierre Nora’s critical concept “lieux de mémoire” aligns here “with the sense that there is no spontaneous memory, that we must deliberately create archives, maintain anniversaries, organize celebrations, pronounce eulogies, and notarize bills because such activities no longer occur naturally.” ^52^ In this context wedding media can be seen as an archive which is no longer spontaneous. Nora describes it as an outside memory that the less “is experienced from the inside the more it exists only through its exterior scaffolding and outwards signs […].” ^53^ Nora refers to archives like the ones in museums where the visitors mostly practice cultural memory. Sometimes individual memory is also activated, in instances where the visitor relates biographically to the exhibition.

In the current study the spaces of memory of wedding photography can be understood as cultural archives that include an individual and collective dimension as previously mentioned. At the same time the spaces present different moments during which memory is created. Therefore, the analysis of the memory process is systematized into four spaces that include certain practices. (1) During the wedding the production of wedding media is understood as one memory space where even pre-wedding shoots take place. (2) The second space is constituted by the photos and videos themselves as the memory space of representation. They follow or play with certain aesthetic norms of wedding depictions. Photos are presented sometimes in photo albums, as a pack of unsorted photos, or in a digital folder on a hard drive or memory stick. (3) In the third memory space of distribution these images and videos are often shared with friends and families, online or in person. (4) The fourth and final space is the space of reception in which the couple look at the photos and videos. In summary, the concept of memory spaces examines the meaning-making processes of wedding photos and videos by considering their context, raising questions about how they are represented, produced, distributed, and received. ^54^

## Methods

The current study is based on conversations in four languages: German, Italian, English, and Swiss-German. Twenty heterosexual and seven homosexual couples were interviewed in Germany, Italy, and Switzerland between April 2022 and February 2023. Ten couples married in a civil ceremony, six of whom are same-sex couples. Only one gay couple married in a traditional religious ceremony. The sample includes seventeen traditional religious and ten alternative ceremonies that took place between 1968 and 2022. Among the 54 participants 24 participants are female and 30 are male, by self-report regarding sex. ^55^ The aim was to meet married couples in three different countries, of diverse cultural and religious backgrounds, different sexual orientations, and different years of marriage, to achieve a diversity of political, cultural, and religious contexts of social actors. This diversity of contexts sharpens the study’s focus on the media and how people remember the event. The procedure allows for a better understanding of the commonalities and differences between memory processes in media practices.

The semi-structured conversations were recorded with a camera applying the method of the video conversation that is rooted in visual anthropology. ^56^ The semi-structured conversations were video recorded with an Iphone 13 max, a tripod, and an external microphone that was attached to the Iphone. Most of the meetings took place in the participants’ homes or apartments. The couple sat next to each other looking at the wedding photos. The setting was arranged so that both the couple and the photos could be recorded in the same frame. During the conversations the social actors were observed by the camera as they looked at and commented on their wedding photos and videos. This method refers to photo-elicitation of which a variety of applications are available. ^57^ In the current research project “reflexive photography” was applied, meaning, couples were asked to tell their wedding story by showing and commenting on their photos. There was some variability in the structure of the conversations because the weddings differ. Some couples got married in a civil service whilst other weddings took place in a religious ritual. In Italy, for example, civil and religious marriage can be done in the church where the priest gives the sacrament and acts as registrar. This is not possible in either Switzerland or Germany.

The conversations were structured in three parts: the first part was about the preparation, the second about the wedding itself, and the third about the meaning of their wedding media. The couples were asked, for example, about the role of their wedding photos and videos in remembering their wedding. Examples of questions asked include (for the full surveys, see *Extended data* ( Mäder, 2023w)): On what grounds did they chose the photographer? Which is their favourite photo of their wedding day, and why? What was the most important moment? What would you change in retrospect if you could? When did they last look at the photos, and are there particular occasions when they have looked at them? However, for most of the conversations, a question-and-answer format wasn’t applied but rather the couple recounted their own wedding narrative.

Methodologically, the camera as a means of collecting data was crucial. During the subsequent analysis that has been conducted with the software atlas.ti (version 23.2.0) the recordings allow the viewer to see which photo the couple was talking about. ^58^ Additionally, the emotional reaction to the images are not only expressed by words but also by the couple’s body-language. Finally, there are many moments in which nobody is talking but there are still reactions to the photos and interactions between the couple which are then caught by the camera. The video protocols are transcribed verbatim ^59^ and evaluated by combining grounded theory and sociological hermeneutics of knowledge. ^60^ The analysis focused on the way the couple comments upon and emotionally responds to the photos and videos of their wedding, how they construct their wedding narrative with the help of these media sources, and if specific patterns related to gender could be observed during the conversations. Additionally, the representation strategies of the wedding photos and videos are analysed and compared.

## Findings

The three memory spaces of production, representation, and reception structure the following analysis of the conversations with the couples. The space of distribution is included in the reception space because the way in which the photos and videos are accessible and distributed overlap. Looking at the photos or videos requires that they had been distributed. Additionally, making wedding media available is based on a modality of distribution. In the space of production, the focus is on how the couples remember the photographer and the taking of pictures during the wedding. In the space of representation, the photographs and videos are analysed scrutinizing wedding photography standards. Finally, in the reception space, the couple’s reactions and comments on the photos and videos are summarized. In all four memory spaces the “sticking power” of memory performance is achieved through emotions. Therefore, the analysis of the conversations focuses on the emotional experiences, how memory is performed, and how it achieves its “sticking power” within these spaces. ^61^

### Memory space of production

**Figure 1.**
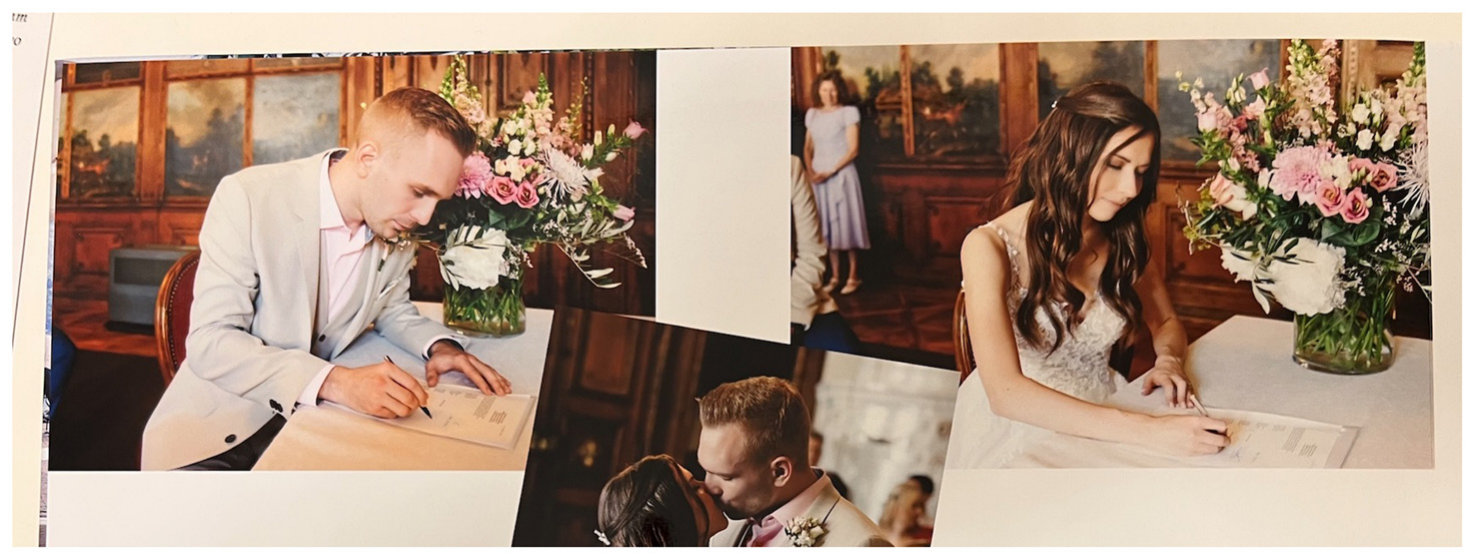
Civil ceremony in Switzerland 2022 (photos in an album, VIW_22_CH_010223_PH06).

Ten couples didn’t hire a photographer for the wedding whilst seventeen hired a photographer or a professional photographer was already known to them, among their friends and family. But even in cases where there was no professional photographer, the photographing and posing for pictures still played an important role. During the conversations there were two different perceptions on the photographic process during the wedding. There were moments in which the photographer wasn’t noticed. This was especially during the ceremony when many couples didn’t realize the presence of the photographer. In some cases, the photographer provided instructions before the ceremony that the couple should slow down or even freeze for a moment in order to be able to “catch the important moment”. This was for example the case during the signing of the wedding contract or the exchange of rings as one husband explained ( Figure 1):

Man: The only moment I really paid attention to him was there. And also there. Because he told me, before you sign, just slow down, just wait with the pen. Because many do it far too quickly and then it’s gone.
Woman: And then you don’t have a photo of it at all.
Man: He said the same thing [like during the signing] to me while putting on the ring. “Pause for a round when you’re there. And try not to cover it with your hand but try it nicely. Then there will be a good photo of it too.” I tried to think of these things. But otherwise, I didn’t notice him taking any photos. ^62^

The example shows how the taking of pictures can become a part of the ceremony by influencing its procedure. The couple naturally and without questioning followed the photographer’s instructions to ensure they have a photograph of this important moment. The ceremony itself is being altered through this. But the couple play the game by pretending that freezing is part of the ceremony. They perform for the photographer to receive in return the pictures as a memory of this central moment of the ceremony.

**Figure 2.**
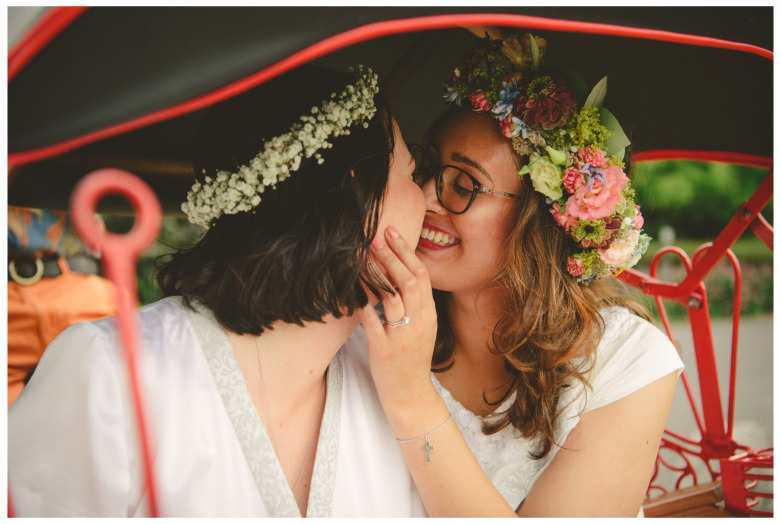
Private moment after the ceremony of a partnership registration in Switzerland 2019 (VIW_12_CH_171022_PH04).

There are other important moments that most want to be captured photographically. There is the kiss after the ceremony. Usually, there is no photo of a kissing couple before the ceremony. The photo of the kissing newlywed couple exists in almost every wedding photo collection ( Figure 2). The post-wedding kiss has a different quality to the pre-wedding kiss because of the ritual transformation from single to couple. This social transformation cannot be captured photographically. The image of the kiss refers to love, affection and legitimate sexuality. It therefore becomes a proxy for the change in social status.

**Figure 3.**
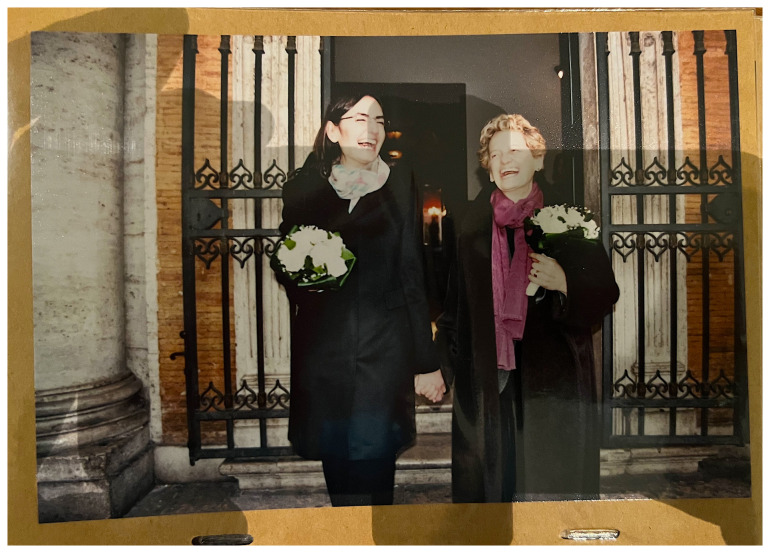
Photo of the second exit after a partnership registration 2016 in Rome/IT (photo in an album, VIW_26_IT_110223_PH02).

Another important moment to be photographed is the exit from the ceremony venue. These photographs are dynamic by nature. In the wedding video of two women who conducted their civil union in Rome in 2016 at the registry in Campidoglio reveals how willing the couples are to follow the instructions of the photographers. ^63^ The couple actually didn’t hire a professional photographer, but friends took the photos and gave the wedding album to the couple afterwards as a gift. Almost at the end of the wedding video the couple exits the civil registry office and the guests are clapping. Suddenly the couple turns around and re-enters the building to exit again ( Figure 3). There is a voice who recalls them “Piano, piano!” meaning they should exit slowly, to carry out the role better the second time. One wife explains: “They had told us we had left too fast and they hadn’t taken any pictures.” The other wife adds: “I have to say that we laughed a lot.” ^64^ The video includes this double exit as a funny moment to be remembered. During the conversation both women were whole heartedly laughing about this memory that obviously achieved sticking power.

**Figure 4.**
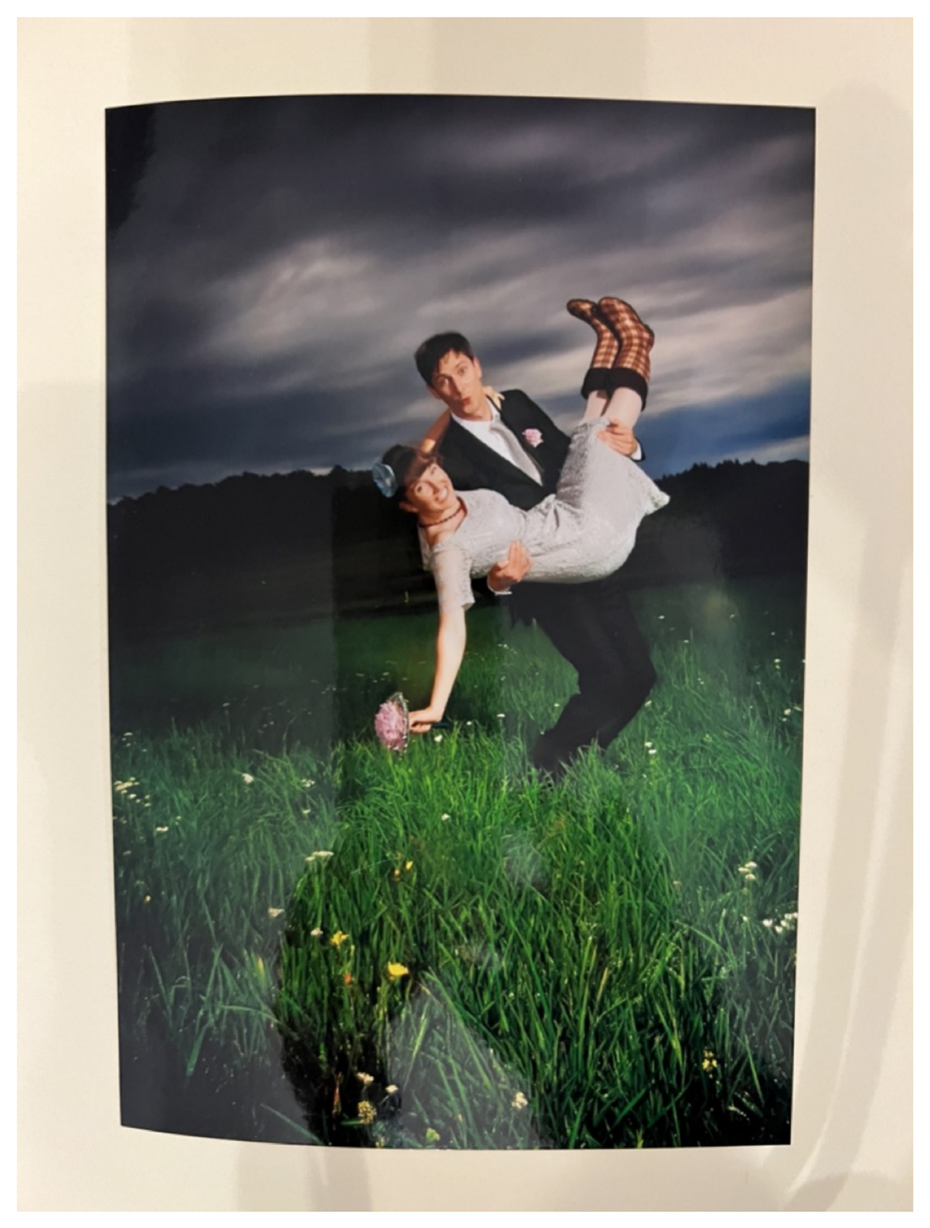
The favourite photo with rubber boots at a wedding in middle Germany 2011 (VIW_07_DE_160923_PH03).

It was striking that the couples often told stories about the making of the pictures. They remembered the shooting as a special and exciting moment with some almost miraculous coincidences. The wedding shoot of a German couple who married in 2011 close to Frankfurt/M took place after a thunderstorm ( Figure 4). ^65^ The professional photographer returned in the evening to take some pictures of the couple, the best man and the maid of honour. The couple remembers the shoot vividly and they enthusiastically told me how it went:

Woman: And at seven o’clock the photographer came and then asked: ''Should I come again tomorrow? Do you want to get dressed again tomorrow or do we want to take pictures afterwards?" And then we said: “We’re going to take pictures now. We’ll get rubber boots.” [Laughter]
Man: Yes, the rubber boots, we weren’t the only ones who ruined their shoes – I think – the whole wedding party did.
[…]
Woman: It’s like, yes, well, that’s a meadow that was particularly great because it wasn’t mowed, but it’s a place like that, I really like being there because you can see so far and we have also said: “We don’t want to go back to the castle somehow [where we married] to take photos'”, but somehow a place that – maybe for me – has a meaning and you thought it was good too? ^66^

The couple agreed on all the details, the atmosphere, and how they felt emotionally about the photo shoot even though the wedding took place twelve years ago. When looking at the series of portraits on this meadow and speaking about the thunderstorm and the rubber boots that they wear, their enthusiasm was obvious and their overlapping recollection striking. They laughed a lot about their story although the wife was more engaged. The husband showed his approval with a guarded smile. The photo with the rubber boots had been sent as a Thank-you card to the guests and the grandmother chose that one too instead of the serious one with the wedding shoes.

Here the photo shoot became an important moment to be remembered and perpetuated in the “rubber boots photo”. By its reception and the couple’s vivid memory of the photographer’s first gaze, the picture achieved what Belting describes as “permanence”. ^67^ The photographer’s gaze through the lens of the camera is inscribed in the photo. When the couple is looking at the picture the image achieves a permanent quality beyond this distinct moment of the act of photography. Additionally, the act of remembering the emotions during the photo shoot provides “sticking power” for the couple.

In conclusion to the memory space of production, the following observations can be made. The phenomenon of sticking power manifests itself during the production process, particularly during the creation of the image, and is subsequently activated during the reception process. In this context, memory refers to the specific moments at which the photographs were taken. Upon viewing the photographs, the memories of the often amusing and jubilant photo sessions are reactivated. It is not uncommon for the emotional moments of a wedding to remain present in the absence of photographic documentation. Conversely, the act of creating the image would be inconsequential if it were not subsequently recalled by the image itself. It is evident that memories of the act of taking pictures are contingent upon the act of taking pictures itself. The mediatisation of memory in the production space does not entirely alter the recollection of the wedding; however, it does extend it through the additional experience that can be remembered. The act of taking photographs, and not merely the photographs themselves, thus serves to reinforce the ritual in question and the memory in retrospect.

### Memory space of representation

The memory space of representation includes the actual photos and videos that have been taken. There are different ways in which the photos are presented. Some couples owned an elaborate wedding album with additional drawings and ornaments. One couple just brought their wedding photos in envelopes and apologized that the photos haven’t been pasted into an album. There were seven couples in all that didn’t have a photo album. Only four couples had a professionally produced video, one of them wasn’t pleased about the result, so they didn’t want to show it. Three other couples owned a video that was shot by friends or family members. The couples who had a video were married at different times and in different countries. One of the couples with the professional video had additionally saved some images on their cell phones that had been posted in social media channels. Three couples only stored their photos digitally and apologized for doing so.

In the conversations the wedding album as a standard to present the photos has been positively evaluated. If there is no wedding album, the couple often apologizes because photo stacks and digital images are perceived as less valuable. According to a wedding photographer, digital photos often get lost. This is why he always strongly recommends couples to produce a wedding album that doesn’t get lost. ^68^ Even if there was a great variety in how the photos have been stored, when presenting them the ritual structure of the images has always been respected. ^69^ There was even a great urge to remember their wedding in chronological order related to the attempt to recreate that time. In all cases of the heterosexual couples, the wife was more invested in the production of the wedding album. In the following section, some cases are highlighted that show the importance of presenting and appreciating the photos appropriately.

A young Italian couple, married in 2022, showed their “provisional” wedding album. The wife collected the pictures that their friends sent her during their honeymoon. She didn’t want to wait “half a year” for the photographer’s official and professional wedding album. ^70^ The woman uploaded their friends’ cellphone photos to an online platform and arranged them in an online tool that provides software to compile albums. The albums can be ordered as print outs. She looked at these photos during the whole honeymoon. On their way back, she compiled the album.

**Figure 5.**
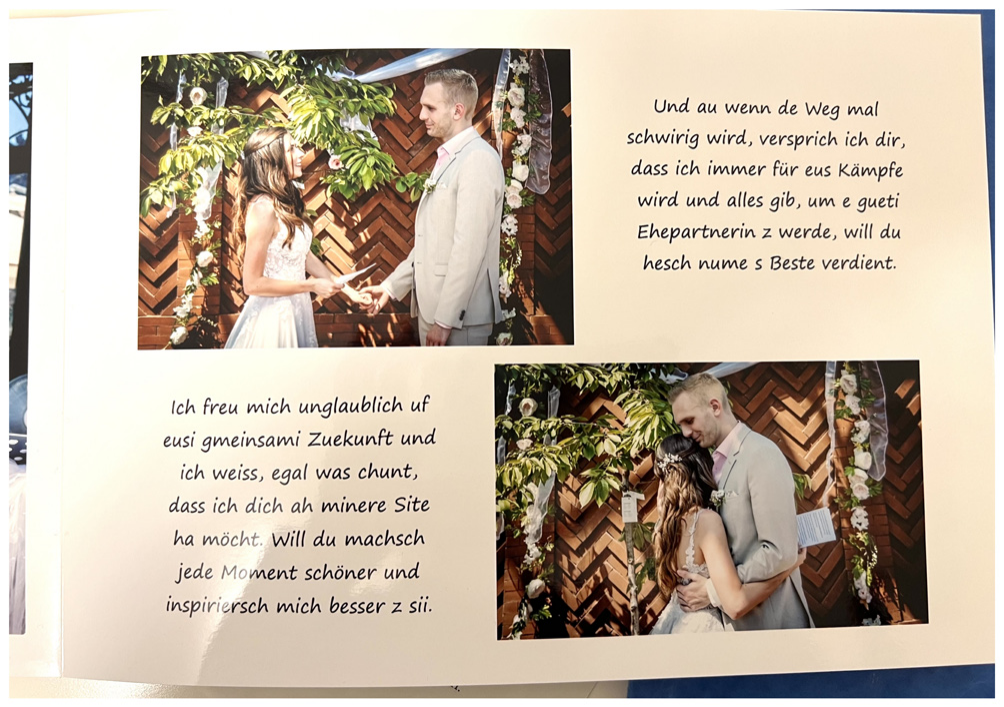
Album of an outdoor wedding ceremony in Switzerland in 2021 (VIW_22_CH_010223_PH07).

Another younger couple’s album contains a lot of texts ( Figure 5). These texts had been transcribed by the wife from the two videos of the wedding. The album begins with the story of their engagement that is written by the husband. ^71^ The photo album is extremely elaborated, and it is obvious that the woman invested a lot of time in compiling the photos and transcribing the texts.

The couple married in three ceremonies. The first was at the registry office ( Figure 1), the second in a member’s garden house ( Figure 5) who blessed them in a self-designed wedding ceremony that was “like in a film as my wife wanted” as the husband commented. ^72^ The couple are members of the Church of Jesus Christ of the Latter-day Saints therefore the third ceremony took place in the temple in Zollikofen in Switzerland. Photography and filming are strictly forbidden in the temple. So, the “filmlike” garden ceremony could be seen as a kind of proxy ceremony to be photographed to replace the temple ceremony. ^73^

**Figure 6.**
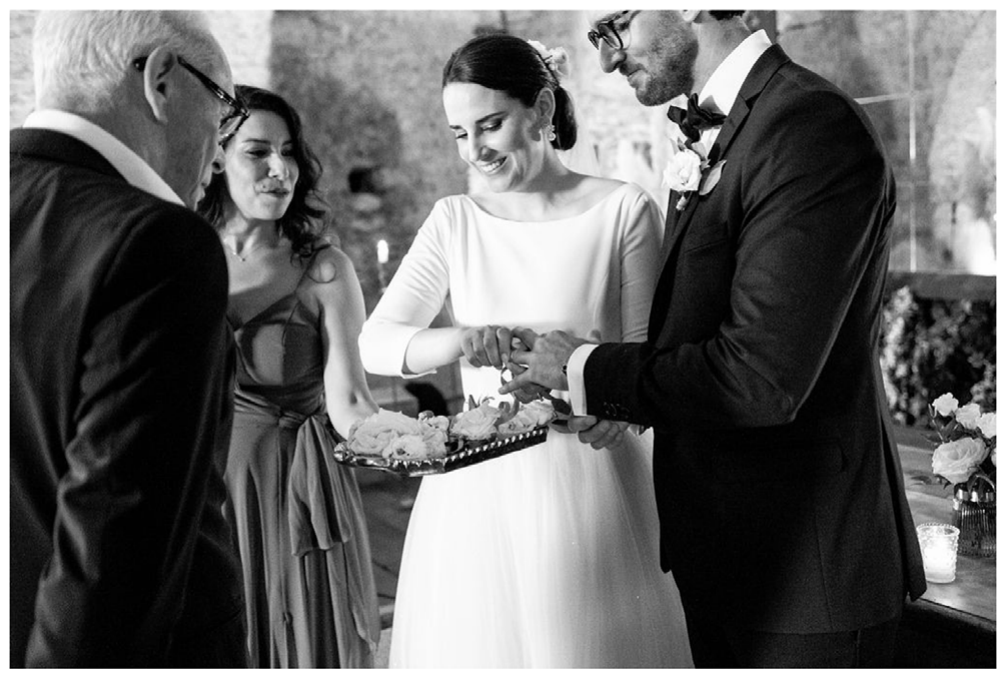
Alternative wedding ceremony in North Italy in 2022 (VIW_15_DE_311022_PH01, photo: Andrea Gilberti).

In general, it can be stated that the ceremony itself is very densely depicted compared to other parts of the wedding. The style of the reception doesn’t matter, there are equally as many photos of an alternative ceremony as of a traditional religious or a civil ceremony. For example, there is one couple who married in an alternative ceremony that took place in the wine cellar of a wedding venue ( Figure 6). The place is reminiscent of a crypt. The wedding started at 5pm and lasted until midnight. The ceremony with a master of ceremony who was the husband’s cousin took about 45 minutes. From their 478 photos selected by the photographer 97 depict the ceremony, specifically the ring ritual that according to the husband refers to a Turkish engagement ritual. The husband is half Turkish and his wife is Swiss and they live together in Germany. Also, for this wedding the wife admitted that she felt much more in charge than the husband.

**Figure 7.**
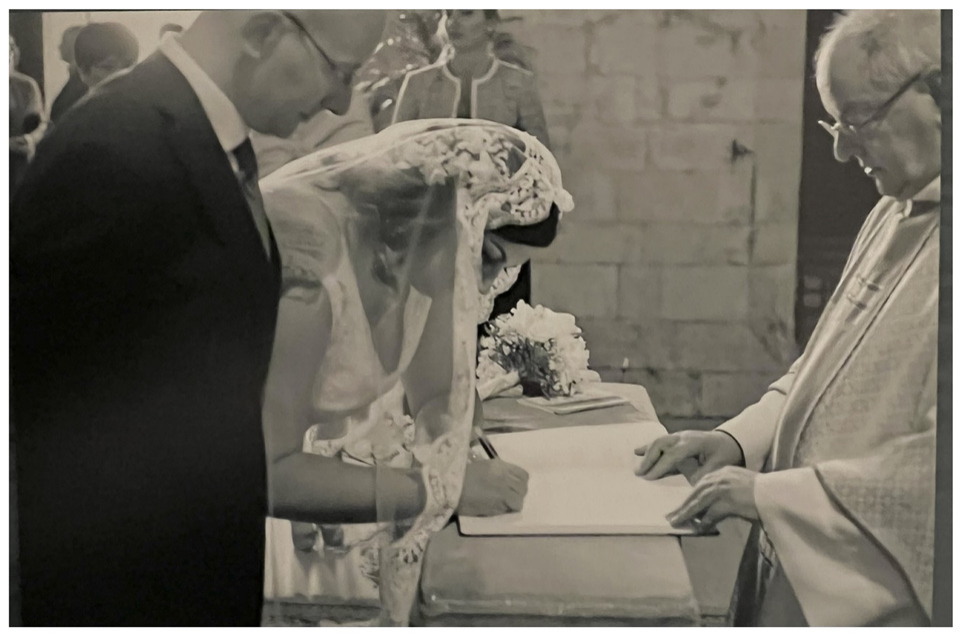
Photo of a Roman-Catholic “rito disgiunto” combined with a civil wedding in middle Italy in 2014 (VIW_09_IT_270922_PH01).

Often when the couples are asked to choose one of their favourite pictures, one is from the wedding ceremony. Even the couple who celebrated the wedding in the Catholic Church in a “ *rito disgiunto*”, which means according to the couple that one person receives the marriage sacrament and the other doesn’t ( Figure 7). Both agreed that they liked the photos in the church during the ceremony the most.

**Figure 8.**
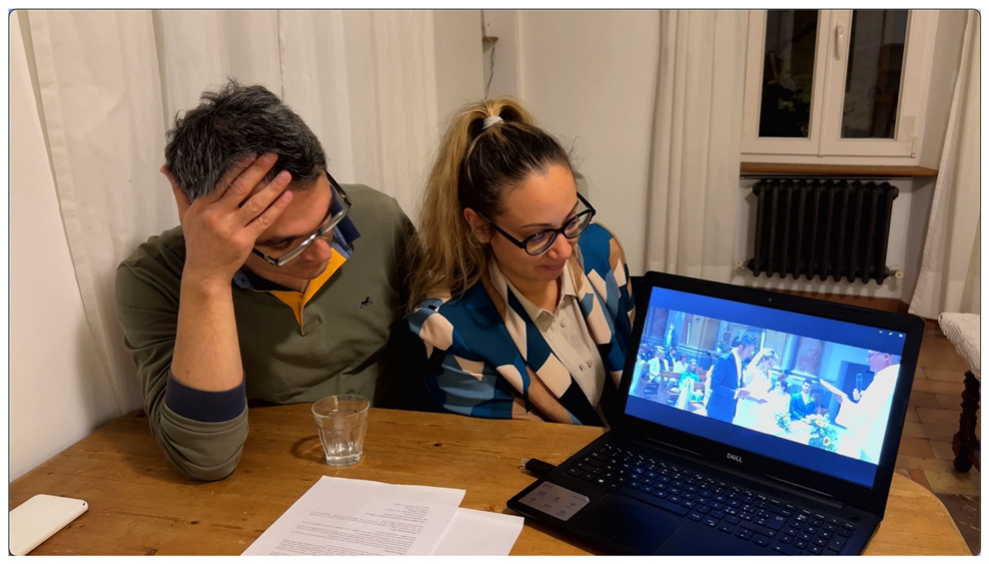
The couple watches the video of their wedding in middle Italy in 2022 (VIW_11_IT_290922_IV_still001253).

An Italian couple who showed their professionally produced wedding video commented a lot while watching the video ( Figure 8). ^74^ But they stopped talking when the Catholic ceremony in the church with the priest was played. The wedding video is about 21 minutes long and the ceremony is shown for 8 minutes which is in a blatant temporal disproportion to the rest of the wedding celebrations.

Considering that the wedding started at 11 am and ended close to midnight, the ceremony of less than an hour was only a small temporal part of that day. In the reception, the party with the guests, becomes less important and the official ceremony occupies a prominent place. Furthermore, in this case, the wedding ceremony has been highlighted in the wedding video and influences also how certain moments of the wedding are selected and remembered. The same could be observed in the conversations. Because there were so many pictures of the ceremony, the conversation dominantly revolved around this topic.

The depiction of the ceremony is also highlighted in the civil registry office as in the case of the lesbian couple who married in Rome in Campidoglio. ^75^ They received an offer from a videographer who filmed the ceremony to buy the video afterwards which they accepted. The video highlights the same moments in a similar visual style as in traditional religious or alternative marriage: it shows the speech of the master of ceremony, then the vows of the couple, the exchange of rings, the signing of the contract, the kiss after the wedding ceremony, and the exit from the civil registry office ( Figure 3). And finally, there is a kind of a “victory photo” after the ceremony in almost all cases independent of what kind of ceremony it has been.

**Figure 9.**
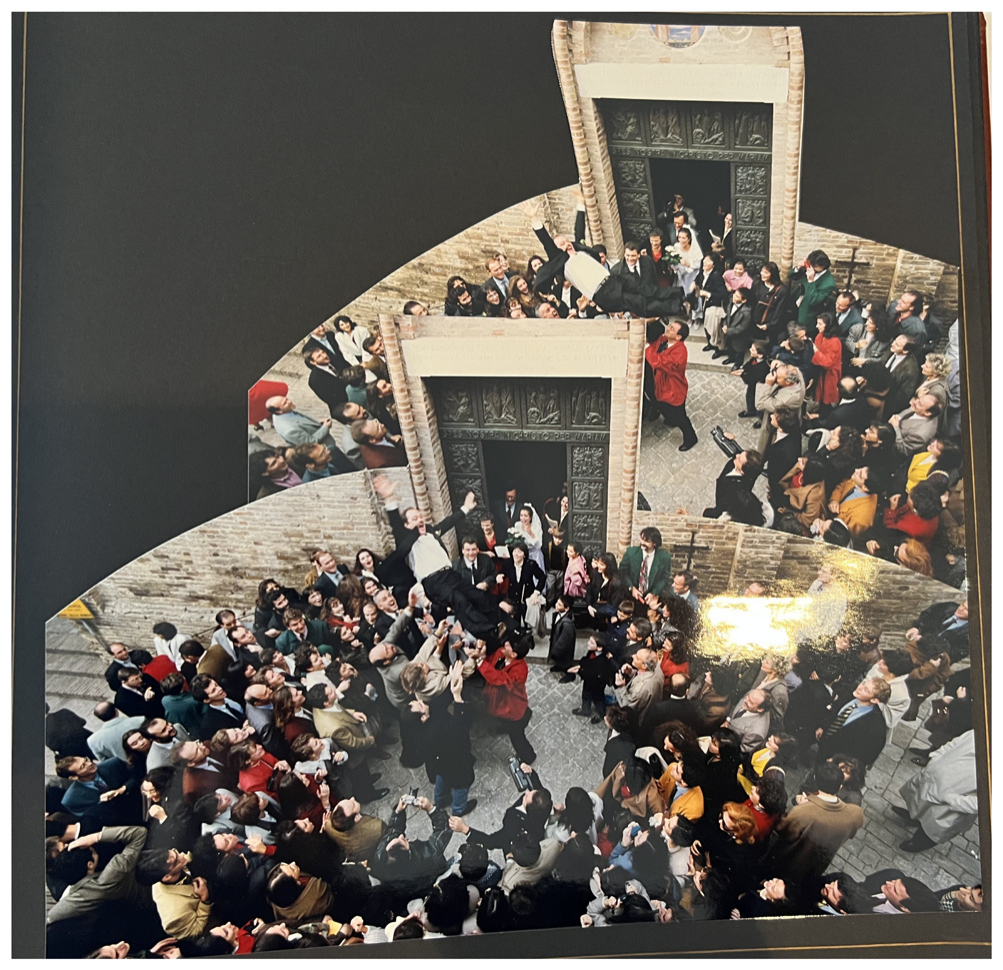
The husband is celebrated like a champion after a Roman-Catholic wedding in middle Italy in 1991 (VIW_02_IT_070422_PH05).

The “victory photo” shows the couple usually holding hands and uplifting the other’s arm as we know it from sport when the winner celebrates his or her achievement. Or as in the current case the newlywed husband is thrown into the air by his friends in an artistically arranged aerial view with two photos ( Figure 9). The photo is often very dynamic and cheerful so that the couples usually like it a lot and choose it as one of their favourite photos.

In conclusion, the memory space of representation can be characterised by a focus on the ceremony regardless of whether it is a traditional religious wedding, a civil wedding, or an alternative rite. The stylistic character of photos and videos do not fundamentally differ. The mediatisation of the wedding collides the religious with the secular sphere. There are, on the one hand, cross-cultural similarities in the materiality of a wedding consisting of “component[s] in the signifying chain”. ^76^ On the other hand, wedding photos stylistically follow the same norms regardless of the cultural-religious background of the couple because “the photographs index something - the image they present is constructed and composed according to cultural codes.” ^77^

Wedding photos themselves have a status which needs to be respected. It does matter how they are presented. Their materiality is appreciated in elaborately designed albums and high quality photo prints often in black and white to make it look more eternal. The photos are not a mere reference to the event but they give the memories a glossy finish. ^78^

### Memory space of reception

The space of reception in which the couple looks at the photos and videos, plays a special role in the research process. First, it is reproduced during the interviews. Often there was an idea that there were different perceptions of the day, but these perceptions could not be in opposition to each other. Therefore the couples tried to actively synchronize their memories during the conversation. Further, the couples, specifically the ones who married in a traditional religious ceremony, wanted to explain in detail the rite and highlighted its significance like the Swiss couple who married in Mumbai in a Hindu ceremony, ^79^ the liberal orthodox Swiss couple in the synagogue in Basel, ^80^ the Italian couple with the Greek orthodox ceremony in Venetia, ^81^ and two more Italian couples who married in a traditional Roman-Catholic rite. ^82^ The husband of one of these Roman-Catholic weddings explained that they belong to the conservative strand “Communione e Liberazione” (CL) and that is the reason the ceremony was specifically significant:

It was a great thing for everyone. How exceptional the ceremony was because the ceremony was truly significant. Six witnesses, three mine and three her’s. Six friends I wanted to have. My sister who read. Another five priests who celebrated. Yes because we experience that – it is strong catholic. […] So you understand that they [the guests] were all a little surprised when they attended this very religious but significant ceremony. ^83^

The couple was equally proud of their Roman-Catholic heritage and the symbolic dimension of the wedding which the photographer sought to depict in the style of the photos. The husband reflects on the quality of the photos:

But then it was nice because if you see the photos, you’ll see that the photos, which are the same thing that strikes those from what you’ll see, is that he made us this beautiful catalogue [photo album] where, however, we are not always in closeup, not with our faces. But there is the context, the friends, the church. Sure, he photographs a part of the church. So the catalogue isn’t just the bride and groom, they are part of a community, of a structure and then at a certain point they also show us. It also shows us– am I saying too much? ^84^

It was striking that the husbands of these couples who married traditionally were especially invested, not only in explaining the ritual wedding procedure in detail, but also in clarifying their wives’ statements or sometimes even correcting them if they thought that she didn’t explain something precisely enough.

Another recurrent topic in the reception process were discussions about how frequently the photos and videos had been viewed. Many looked at their photos with friends and family members after the wedding to remember the wedding day. Other couples share the pictures with their guests. One couple even told me that they have displayed pictures of other weddings but none of their own. There was only one couple who told me that they hadn’t looked at their enormously large wedding album for 27 years. And it bothered them that their daughters didn’t want to look at it. However, they were eager to share their wedding album during the conversation, which was extremely detailed, featuring many oversized photos and a strong emphasis on the ceremony. ^85^

Mostly there is one person who is more enthusiastic in looking at the pictures. Specifically, in the case of the heterosexual couples, it is the woman who talks more about the photos as the example of the German couple shows. ^86^ The husbands often explained the meaning of the symbolic practices that had been depicted in more detail.

Whilst watching the videos and looking at the photos with the couples, some couples explained that these photos look romantic or meaningful. But they also had a lot of fun during their celebrations as this Italian wife remembers:

I remember my wedding, I had so much fun, I always danced, always, I never stopped. And I remember it being really funny. The video on the other hand gives me more of a romantic feeling, like there’s – all these romantic scenes, right? With this cheesy music and so on. So maybe the video made me - I mean it makes me relive the romance part, but I remember it a lot more [laughter], a lot funnier. ^87^

Additionally, the couples mentioned that the photos and video allowed them to see and remember things they didn’t perceive during the day because they were so busy and distracted.

One last observation is that almost all the gay couples expressed sadness and responded emotionally to the photos or the video. Some of them even cried. Their experience of being excluded from this ritual left emotional traces that came to the surface during the conversations. ^88^ In general during most conversations the viewing of the album together was an emotionally intense and personal experience. The couples often appreciated the opportunity to tell their wedding story and sent thanks after the interviews.

The memory space of reception reveals differences between husbands and wives of heterosexual couples but also similarities between heterosexual and gay couples, such as the regularity of viewing the photos. Some couples look at the album regularly or at least once a year. The closer the wedding date is the more often they look at the photos. In general, the husbands of heterosexual couples were more talkative when married in a traditional rite. In these cases, the personal memory has been partially replaced by the explanations of the symbolic meaning. In general it seems easier for wives to capture the event in words, because they often take the lead in the conversation.

The romantic photos were not the ones the couples enjoyed the most. It seems that they were mainly taken to comply with the norm. A random picture, a snapshot of an intimate moment or during which something special happened, often during the ceremony, is mostly named as the favourite picture of the day. It holds an important place in the memory of the wedding. Additionally, the photos reveal moments and things that have been overseen during the celebrations. The photo camera than “augments reality” and the photos become an “ocular prosthesis” ^89^ according to Florence Maillochon. ^90^ This augmented reality is than synchronized when recalled together. In the process of remembering the partly different experiences of the wedding day synchronize. The feelings that arise during viewing the photos combined with the emotional conversations about the photos provide sticking power to the memories that thereby become permanent.

## Conclusion: the mediatisation of the wedding memory

This study about the mediatisation and the memory of weddings considered the role of wedding media such as photos and videos in remembering the *rite of passage*. The twenty seven conversations with couples from Germany, Italy and Switzerland confirmed that wedding photos and videos are highly standardized representations. The memory of their production and reception experiences shows surprising similarities between the couples, independent of the couple’s age, year and country of wedding, sexual orientation, or religious-cultural background. The conversations revealed how the mediatisation of wedding memories achieve “sticking power” through emotions. In the concluding section the following most significant results from the spaces of memory and their relation to each other are reviewed. The thematic areas cover the following topics: (1) The memory of the photo session with the photographer during the wedding day; (2) the photos of the ceremony and their reception; (3) how the media enlarge the wedding experience; (4) the homogenization of memory (and standardisation of emotions); (5) gender differences in the memory space of reception.

(1) In the memory space of production, the importance of taking the pictures was striking and how much time many couples invested or at least took some time alone with the photographer. These photos often have a “romantic style” that highlight a tender and close connection between the couple in a warm light. These depictions aim to highlight an emotional quality of the moment. They show the kissing couple, the couple in the sunset, in a fancy car, in a park with lots of natural surroundings or after the ceremony in front of the registrar’s office, the church doors, or wherever the ceremony took place. The wedding photo session is often experienced as a special event with the photographer and becomes an emotional and even intimate part of the *rite of passage* that is captured in the different photo motifs. Nevertheless, these posed couple photos are often less appreciated than the memory of their making.

(2) The photos of the ceremony play a rather different role in the memory process. The ceremony is conspicuously, densely photographed be it traditionally religious, an alternative rite, or a civil wedding. The standardized and large number of images like the exchange of the rings, signing of the wedding contract, or the “victory photo” of the couple after the wedding highlight the wedding ceremony in the couple’s memories. The emphasis on the ceremony photographically is particularly evident during the conversations, in the memory space of reception, where the ceremony is often extensively discussed. This extensive discussion is due not only to the multitude of photos or the extensive video footage of the ceremony, but also because the ceremony is regarded as an important moment by the couple. Not all of these photo moments are specifically emotional but, in their reception, an emotional value and significance has generally been added. In this way the key moments of the wedding are expressed in the quantity of the photos or length of video footage. In addition to this, the couples often chose the photos of the ceremony as one of their favourites. It shows that both the motifs and the quantity of pictures reinforce a positive value and provide “sticking power”. Both influence how the rite of passage is remembered in retrospect. It is notable that the couples who only owned digital photos of their wedding apologized for this. While couples with elaborate albums were proud to show this “piece of art”. The couples who remembered their wedding with the photo album were often clearer and surer about details than couples without an album or who only had printed photos in a stack.

(3) The memory space of reception enables the couples to see and experience moments of the wedding day they missed out, as one couple explicitly mentioned. The couples often mentioned that they felt a lot of tension during the day and that the day went by very quickly. In this case, the wedding media enables the couple to not only remember but also experience the ceremony or the party from an insider observer perspective. In this way, media enlarge the wedding experience and in the words of Belting “achieve permanence”. Photos and videos transcend the rite of passage to transform it into a consistent value that in the reception process is experienced as confirming and self-assuring. From a mediatisation perspective it could even be argued that looking at the photos and videos allows couples to repeat and revive the intended emotional quality of their wedding. By doing so, the couple’s wedding promises are reinforced in the reception process.

(4) Because wedding images are highly standardized, the memory of the event is homogenized. This homogenization could be observed in the individual conversations. The couple created the wedding narrative together and adapted their different experiences to each other to create one memory. But also between the couples, remarkable similarities could be observed in their memories, independent of religious, cultural, and educational background, or the age of the couples, as already mentioned. This homogenization becomes part of a collective memory of the rite of passage that connects the individuals. Additionally, it strengthens the feeling of belonging to a community (of wedding couples) that transcends the cultural-religious identities.

5) The conversations revealed insightful gender differences in dealing with the wedding photos between (a) husband and wife, (b) in traditional religious weddings, and between (c) gay and heterosexual couples.

(a) In most heterosexual couples, the wives were more invested, be it in compiling the photo albums, looking at them, or talking about the wedding. During these conversations, the husbands often had to be explicitly addressed, to find out more about their opinion and their experiences during the wedding. The wives showed more passion for the photos and felt more in charge of their display and preservation.

(b) In the case of traditional religious weddings, however, the husbands often took the lead in explaining how the religious ceremony is structured. They talked about the symbolic meaning of the different practices, readings, blessings, or props. In cases in which the wife explained the ceremony, the husbands often added details or even corrected her which was seldom the case the other way round. The husbands of these exclusively heterosexual marriages showed fewer emotions. They applied a more educative and rational approach to talk about their wedding. The memory was less about the personal experiences of the wedding day than about the wedding norms of the corresponding tradition. Nevertheless there was one husband, a member of The Church of Jesus Christ of the Latter-day Saints who was very emotional in the recollection of the ceremony, without any photos, as they are not permitted by the church during the ceremony.

(c) The gay couples tended to share their talking time more equally and took turns in telling their wedding narrative. It seemed that gay couples are equally invested in the pictures, and the images of the wedding itself and the reception, was something very emotional and of high importance in a different way than in the case of heterosexual couples. While the heterosexual couples often expressed the view that their wedding was a bit “different”, when looking at the photos they often seemed quite traditional. The gay couple’s wedding was different because they belonged to the first generation who were legally allowed to marry. The political dimension is an important value in the rite of passage of gay couples. One husband mentioned that the difference between a registered partnership and being married always felt like a forced outing: “When I got married, I thought it was bad in retrospect that all the official forms were changed so slowly. Then there was single, married, partnered. And if you ticked partnered, then it was clear that you were partnered with your same-sex partner, because mixed-sex partnerships are not legally permitted. So every time you fill something in, you have to tick the same box and I found that very stressful. So it's also unfair.” ^91^ They often playfully applied traditional wedding themes and adapted it to their lifestyle. Gay cake figures or same wedding dresses express their sexual orientation. The emotionality during the telling of their wedding narrative was often overwhelming.

The study of the mediatisiation of weddings shows that the “sticking power” of emotions takes place in the different spaces of memory. The spaces significantly interact, not only reinforcing but also extending emotions with additional experiences in the spaces of production and reception. Additionally, the wedding images themselves form and even intensify the meaning of the rite of passage and add an emotional charge that in turn keeps memory alive. The conversations about the memory of the weddings guided by the pictures not only scrutinises how the media become a defining part of cultural practices like a rite of passage. But the research also shows how photos and videos may define our memories of cultural practices.

The focus on married couples from different cultural backgrounds, gender, and sexual orientations is at the same time a limitation. The two main commonalities between the couples were firstly, the photos or videos of their wedding, and secondly, that all the couples are still married. Having a conversation with separated couples would have been another way of researching wedding media and their meaning. One separated woman told me that the only thing she will do with her wedding photos is “to burn them”. To include separated couples and widowers could provide an avenue for further research into the topic, and offers the potential for additional insights into the role of media in remembering the rite of passage of weddings.

## Ethics and consent

Ethical approval was granted by the Ethics Committee of the university of Macerata on 22 February 2022 and by H2020-MSCA-IF_2020 (D1.1–D1.5), on 4th July 2022. Written informed consent for publication of the participants details was obtained from the participants.

## Data Availability

Due to protection of privacy and the sensitivity of interview footage, the data can only be shared based on the explicit consent of the participants. The data includes wedding videos and photos in which religious affiliation, sexual orientation and personal details are discussed. It is stated in the written consent form, that the data will not be made open unless the participants agree. The transcriptions of the interviews can be accessed by submitting a request to the author via email, which should detail for what purposes the transcriptions would be used. This project contains the following underlying data: Zenodo: Video Interview with transcript, married couple, Italy https://doi.org/10.5281/zenodo.8248549 ( Mäder, 2023a) Zenodo: Video interview with transcript, married couple, Italy https://doi.org/10.5281/zenodo.8248660 ( Mäder, 2023b) Zenodo: Video interview with transcript, married couple, Italy https://doi.org/10.5281/zenodo.8248820 ( Mäder, 2023c) Zenodo: Video Interview with Transcript, married Couple, Switzerland https://doi.org/10.5281/zenodo.8181259 ( Mäder, 2023d) Zenodo: Video interview with transcript, married couple, Germany https://doi.org/10.5281/zenodo.8186799 ( Mäder, 2023e) Zenodo: Video interview, married couple, Switzerland https://doi.org/10.5281/zenodo.8249679 ( Mäder, 2023f) Zenodo: Video interview with transcript, married couple, Switzerland https://doi.org/10.5281/zenodo.8248393 ( Mäder, 2023g) Zenodo: Video interview, married couple, Italy https://doi. org/10.5281/zenodo.8190190 ( Mäder, 2023h) Zenodo: Video interview with transcript, married couple, Switzerland https://doi.org/10.5281/zenodo.8247614 ( Mäder, 2023i) Zenodo: Video interview with transcript, married couple, Italy https://zenodo.org/record/8178945 ( Mäder, 2023j) Zenodo: Video interview with transcript, married couple, Switzerland https://doi.org/10.5281/zenodo.8248443 ( Mäder, 2023k) Zenodo: Video interview with transcript, married couple, Germany https://doi.org/10.5281/zenodo.8247717 ( Mäder, 2023l) Zenodo: Video interview with transcript, married couple, Italy https://doi.org/10.5281/zenodo.8190308 ( Mäder, 2023m) Zenodo: Video interview with transcript, married couple, Germany https://doi.org/10.5281/zenodo.8181413 ( Mäder, 2023n) Zenodo: Video Interview with Transcript, Married Couple, Germany https://doi.org/10.5281/zenodo.8190269 ( Mäder, 2023o) Zenodo: Video interview, married couple, Switzerland https://doi.org/10.5281/zenodo.8249632 ( Mäder, 2023p) Zenodo: Video interview with transcript, married couple, Germany https://doi.org/10.5281/zenodo.8247577 ( Mäder, 2023q) Zenodo: Transcript of video interview, married couple, Italy https://doi.org/10.5281/zenodo.8190245 ( Mäder, 2023r) Zenodo: Video interview with transcript, married couple, Italy https://doi.org/10.5281/zenodo.8249246 ( Mäder, 2023s) Zenodo: Video interview with transcript, married couple, Italy https://doi.org/10.5281/zenodo.8246842 ( Mäder, 2023t) Zenodo: Video Interview with transcript, married couple, Switzerland https://doi.org/10.5281/zenodo.8247782 ( Mäder, 2023u) Zenodo: Video interview with transcript, married couple, Germany https://doi.org/10.5281/zenodo.8184184 ( Mäder, 2023v) Zenodo: Questionnaires_English_German_Italian https://doi. org/10.5281/zenodo.8249440 ( Mäder, 2023w) This project contains the following extended data: Couples_English_questionnaire.pdf Couples_German_questionnaire.pdf Couples_Italian_questionnaire.pdf Data are available under the terms of the Creative Commons Zero “No rights reserved” data waiver (CC0 1.0 Public domain dedication).
