# Supplementary material for: Promising images of love: a qualitative-ethnographic study about the mediatised memories of weddings
Source: Open Res Eur. 2024 Dec 5;3:198. Originally published 2023 Nov 16. [Version 2] doi: 10.12688/openreseurope.16521.2 (PMC11831601; doi:10.12688/openreseurope.16521.2)
Supplement: Peer review history

# Reviewer report: Reviewer response for version 2

**DOI:** 10.21956/openreseurope.20503.r48171

**Published:** 2024-12-09

**Authors:** Marc A Ouellette

**Affiliations:** 1 Old Dominion University, Norfolk, Virginia, USA

The revisions were very thorough and the author's note was terrific!

If applicable, is the statistical analysis and its interpretation appropriate?

Not applicable

Is the study design appropriate and is the work technically sound?

Partly

Is the work clearly and accurately presented and does it engage with the current literature?

Partly

Are the conclusions drawn adequately supported by the results?

Yes

Are sufficient details of methods and analysis provided to allow replication by others?

Yes

Are all the source data and materials underlying the results available?

Yes

Reviewer Expertise:

Cultural and Gender Studies, with specialization in contemporary popular culture.

I confirm that I have read this submission and believe that I have an appropriate level of expertise to confirm that it is of an acceptable scientific standard.

# Reviewer report: Reviewer response for version 1

**DOI:** 10.21956/openreseurope.17835.r39224

**Published:** 2024-08-14

**Authors:** Julia Carter

**Affiliations:** 1 Department of Sociology, University of the West of England, Bristol, UK

This is a very promising paper which aims to contribute to the existing literature on wedding images and the meanings and consumption of these media. In particular the paper focuses on how individuals make sense of and talking about their re-viewing of their own wedding photos and videos some time after the event. In this respect, the paper contributes to literature on visual media and memory-making. I do have some reservations as the paper currently stands, particularly regarding the literature used to frame the study, and the discussion and analysis of the findings.

 The literature review at the start of the paper provides a brief and sweeping overview of some of the available research on weddings. However, it misses a significant amount of literature and that cited is not used effectively to frame the later discussion of theoretical or empirical contributions (both issues noted by the other reviewers). In this respect, the paper does not fulfil the potential that is evidently there in the rich and detailed data. In addition to the literature suggestions provided by previous reviewers I would add Sharon Boden (2001; 2003), Carter and Duncan (2016) and Boyce Kay, Kennedy and Wood (2020) from the UK and Kavita Ramdya (2011) from the USA. Consulting this additional literature may also aid the deepening of the analysis in the findings section.

 In line with the other reviewers, I would like to see a more detailed and analytical discussion of the findings and what these might contribute to our understandings of the construction of meaning around weddings, gender, sexuality, and memory. In particular, the comment on the homogenisation of wedding memories seems really important- is this process limited to the wedding photos or memorisation or is there something wider about weddings more generally that might promote this sense of homogeneity, perhaps in relating to a community that transcends individual identity markers? Taking the analysis of the findings further would really strengthen the paper and its contribution to existing debates.

 References:

- Boden, S. (2001) ‘Superbrides’: Wedding Consumer Culture and the Construction of Bridal Identity. *Sociological Research Online*, 6(1), 1-14. https://doi.org/10.5153/sro.570
- Boden, S. (2003) *Consumerism, Romance and the Wedding Experience*. Palgrave macmillan.
- Boyce Kay, J, Kennedy, M. and Wood, H. (eds.( (2020) *The Wedding Spectacle Across Contemporary Media and Culture: Something Old, Something New*. Routledge.
- Carter, J., & Duncan, S. (2017). Wedding paradoxes: individualized conformity and the ‘perfect day’. *The Sociological Review*, 65(1), 3-20. https://doi.org/10.1111/1467-954X.12366
- Ramdya, K. (2011) *Bollywood Weddings: Dating, Engagement, and Marriage in Hindu America*. Lexington Books.

If applicable, is the statistical analysis and its interpretation appropriate?

Not applicable

Is the study design appropriate and is the work technically sound?

Yes

Is the work clearly and accurately presented and does it engage with the current literature?

Partly

Are the conclusions drawn adequately supported by the results?

Yes

Are sufficient details of methods and analysis provided to allow replication by others?

Yes

Are all the source data and materials underlying the results available?

Yes

Reviewer Expertise:

Sociology of marriage, relationships, weddings and gender

I confirm that I have read this submission and believe that I have an appropriate level of expertise to confirm that it is of an acceptable scientific standard, however I have significant reservations, as outlined above.

# Response

**Published:** 2024-11-22

**Authors:** Marie-Therese Mäder

**Affiliations:** Philosophy, Ludwig Maximilians University Munich, Munich, Bavaria, Germany

I value your feedback and I am grateful for your input. The current state of research has been comprehensively updated to include relevant literature about wedding photography with a particular focus on memory. The literature review therefore addresses the ways in which norms and values are expressed in wedding photography, as well as the extent to which photos and videos influence memory processes. The overview of the discussed literature is structured in the following four areas: (1) norms and values, (2) memories, (3) the ritual dimension, and (4) emotions. As you proposed, the analysis has been further developed by providing a more comprehensive examination, structured presentation, and summary of the findings at the conclusion of each section. The concept of the homogenisation of memory has been further elucidated through an examination of the photographic production and stylistic elements, as well as the reception of these images. The combination of all three moments evokes emotions that provide a lasting impression, or "sticking power," to the memory of the event. The section presenting the findings (formerly titled "results") has been expanded with the inclusion of photographs from the fieldwork, thereby enhancing the clarity of the argument. The conclusion section has been extended with a cross-reading of the analyses results where the relevant point you make about transcending individual identity markers has been included. Your feedback is greatly appreciated! It helped me to work out individual points such as the homogenisation of memory in more detail.

# Reviewer report: Reviewer response for version 1

**DOI:** 10.21956/openreseurope.17835.r39223

**Published:** 2024-05-08

**Authors:** Matthew Wade

**Affiliations:** 1 Department of Social Inquiry, School of Humanities and Social Sciences,, La Trobe University, Melbourne, Victoria, Australia

Thanks for the opportunity to review this study, which is a very high-quality contribution to what remains a curiously understudied practice. Weddings retain such a vaulted status across cultures, and their memorialisation in photographic and videographic forms has only become increasingly complex and professionally curated, creating mementos that have incredible appeal. And yet, there is little research on the hopes and motivations in producing these affecting tokens of memory, which often hold such importance in idealized life narratives. It was a therefore a pleasure to read this study, and I look forward to seeing the final version.

 Echoing the prior reviewers, my suggestions relate primarily to strengthening the literature review and the analytical findings.

 The author states:

 ‘The research question scrutinizes weddings from a contemporary perspective with a focus on the media that are produced during the wedding. These wedding media include photos and videos that are produced professionally, semi-professionally, or non-professionally. About this specific topic, no scholarly literature has been published so far.’

 I must confess I was surprised to read this and even a little personally hurt. As another reviewer has duly noted, there is a small but significant corpus of material specifically on media – both photography and videography – that is produced in memorializing weddings. None of these studies appear to be cited, and the analysis is oddly conspicuous for their absence. Of course, some pragmatic circumscribing is always necessary in a literature review, particularly when dealing with an expansive topic (such as the cultural meanings attached to wedding rituals). However, to not acknowledge prior studies that investigate the precise empirical focus of your own study results in a striking void.

 This study, to my knowledge, is the first to use photo and video-elicitation methods to discuss wedding media with the key actors captured in such events. This is an immensely valuable contribution to the field. However, this originality and benefit is actually diluted and obscured by not acknowledging what has come before. All of the studies below analyze wedding photography/videography in some form, and merit inclusion in the literature review:[ Bezner L.2002 (Ref 1), Lewis C.1997(Ref 2), Lewis C.1998 (Ref 3), Schwarz O.2010 (Ref 4), Strano M. 2006 (Ref 5 ), Wade M, et.al.,2019 (Ref 6), Social Beings, Future Belongings 2019 (Ref 7), Walsh M, et.al., 2020 (Ref 8), Humble Á, et.al., 2008 (Ref 9)]

 I also suspect that engaging with these studies will help with the theoretical framing and analysis of the findings. While the study is highly original and praiseworthy, many of the points raised are echoed in previous work. In particular, regarding the paper’s ‘four memory spaces of production’, there is much prior analysis on *production* (especially how photographers and videographers approach their work in creating something that is uniquely affecting but also adheres to expected conventions) and *representation* (e.g. critical analyses of how rigidly traditional tropes are reproduced in wedding mementos).

 Similarly, some of the findings, while very interesting, tend to *describe* but leave any *analysis* implicit. For example, along with being a memorably comic moment in having to recreate key events, why are couples so self-conscious and committed to capturing very specific aspects for posterity (p.12)? Why does a tangible wedding album seem to hold greater value than a digital one (p.13)? Why do couples take an increasingly proactive and co-creative role in curating their albums and narratives (pp.13-14)? Why does the ceremony occupy such an outsized role in the memoralization of the wedding (p.14). Why, do you think, were husbands particularly active in clarifying symbolic or procedural aspects of the ritual, but were otherwise relatively uninvolved, leaving their wives to primarily continue to carry the ‘wedding work’ of articulating the central narrative (p.15)? Can you connect the particularly ‘emotionally intense and personal experience’ for gay couples with other work that has explored the heightened affective resonance for those once excluded from culturally celebrated rituals of belonging (p.15)?

 [on ‘wedding work’, see [9]. It’s interesting that women in heterosexual couples primarily continue to do the wedding work even years after it has taken place. They appear to take primary responsibility for cultivating the collective memory of the occasion.]

 In short, the findings are very interesting but could benefit from some further analysis and connection to prior work (either theoretical or empirical). It’s strange that once the discussion of results begins not a single further source beyond the interviews is cited in the rest of the study.

 Finally, there are small typographical errors that could be corrected (e.g. ‘of the of the’, p.7).

 Thanks again for the opportunity to review this excellent paper and look forward to reading the final revised version.

If applicable, is the statistical analysis and its interpretation appropriate?

Not applicable

Is the study design appropriate and is the work technically sound?

Yes

Is the work clearly and accurately presented and does it engage with the current literature?

Partly

Are the conclusions drawn adequately supported by the results?

Partly

Are sufficient details of methods and analysis provided to allow replication by others?

Yes

Are all the source data and materials underlying the results available?

Yes

Reviewer Expertise:

Sociology, Cultural Studies, Social Policy

I confirm that I have read this submission and believe that I have an appropriate level of expertise to confirm that it is of an acceptable scientific standard, however I have significant reservations, as outlined above.

## References

[1] : Wedding Photography: ‘A Shining Language’. *Visual Resources* .2002;18(1) : 10.1080/01973760290026477 1-16 10.1080/01973760290026477

[2] : Hegemony in the Ideal: Wedding Photography, Consumerism, and Patriarchy. *Women's Studies in Communication* .1997;20(2) : 10.1080/07491409.1997.10162409 167-188 10.1080/07491409.1997.10162409

[3] : Working the Ritual: Professional Wedding Photography and the American Middle Class. *Journal of Communication Inquiry* .1998;22(1) : 10.1177/0196859998022001006 72-92 10.1177/0196859998022001006

[4] : Negotiating Romance in Front of the Lens. *Visual Communication* .2010;9(2) : 10.1177/1470357210369982 151-169 10.1177/1470357210369982

[5] : Ritualized Transmission of Social Norms Through Wedding Photography. *Communication Theory* .2006;16(1) : 10.1111/j.1468-2885.2006.00004.x 31-46 10.1111/j.1468-2885.2006.00004.x

[6] : ‘Their time and their story’.2019; 10.4324/9781315200859-3 26-42 10.4324/9781315200859-3

[7] Social Beings, Future Belongings.2019; 10.4324/9781315200859 10.4324/9781315200859

[8] : Soundtrack for love: wedding videography, music and romantic memory. *Continuum* .2020;34(1) : 10.1080/10304312.2019.1700216 14-31 10.1080/10304312.2019.1700216

[9] : “The Royal We”. *Journal of Family Issues* .2008;29(1) : 10.1177/0192513X07305900 3-25 10.1177/0192513X07305900

# Response

**Published:** 2024-11-22

**Authors:** Marie-Therese Mäder

**Affiliations:** Philosophy, Ludwig Maximilians University Munich, Munich, Bavaria, Germany

I appreciate your valuable feedback of which almost everything has been implemented. Most of the questions you raised in the review have been addressed in the revision. The method of photo and video-elicitation has been highlighted in the argument. The analysis has been further developed by providing more in-depth analysis, structuring and summarising the findings at the end of each section. The results are synthesized in the conclusion that has been completely revised. The aim was to create cross-connections between the memory spaces of the analysis. The literature review has been completely revised to include relevant literature on wedding photography suggested by the reviewers. It focuses on how norms and values are expressed in wedding photography and in what sense the photos and videos influence memory processes. Most of the suggested literature has been included in the argument, such as Lili Corbus Bezner, Charles Lewis, Michele Strano, both texts by Mathew Wade and Michael Walsh. The overview of the literature discussed is divided into the following four areas: (1) norms and values, (2) memories, (3) the ritual dimension, and (4) emotions. The "Findings" section presents the results of the study and relates the analysis of the memory process to existing studies for confirmation or as an addition to existing studies. The analysis is systematised into the four spaces of production, representation, reception and distribution (the latter two have been combined in the analysis because of their overlapping characteristics), which include certain practices. Each section is followed by a summary of the main interim findings. Photographs from the fieldwork have also been included to strengthen the argument. Thank you for your feedback! It has helped me to make progress on the research. I have managed to link the observations from the interviews more closely to the theoretical framework. The review has also helped to differentiate the results of the field research more clearly. This means the results are now presented in a more effective way.

# Reviewer report: Reviewer response for version 1

**DOI:** 10.21956/openreseurope.17835.r39222

**Published:** 2024-04-22

**Authors:** Marc A Ouellette

**Affiliations:** 1 Old Dominion University, Norfolk, Virginia, USA

While the paper is timely and does a good job presenting its basis, it does provide a notably incomplete view of the existing literature in the field. In particular, I have listed 3 articles that would seem to cover many of the same issues raised in this article, which makes them even more noteworthy for being absent. In particular, I take issue with the following statement from the article:

 "The research question scrutinizes weddings from a contemporary perspective with a focus on the media that are produced during the wedding. These wedding media include photos and videos that are produced professionally, semi-professionally, or non-professionally. About this specific topic, no scholarly literature has been published so far."

 In fact, I would offer 3 articles that cover this ground:[1],[2]

Semiotic Review

 Moreover, the second of these, Kimport's 2012 look at same-sex wedding photographs has it in the very title of the work and the last of these, Ouellette's 2018 piece in *Semiotic Revie*w, considers precisely the media that are produced during the wedding by focusing not only on the production of wedding photographs, but also on the assemblage of the materials. These two articles not only do the work of challenging the culture, the politics, the economy, they do so precisely by studying the media--wedding photographs--produced during the wedding. Thus, the literature review is not only incomplete, it replicates without acknowledging work that has actually been done elsewhere and so it needs to be careful about how it differentiates itself from these earlier studies.

 For its part, the first of the three, Fetner & Heath's consideration of same-sex weddings and the "fairy tale" myth also considers the production of the wedding day itself. 

 Each of these provides a challenge to the ritual (a ritual that is well documented in Austin's speech act, but which also falls well under the rubric of Tamiah's 1985 consideration of ritual. Thus, I find that the article somewhat intriguing but also somewhat disappointing. Moreover, if it is going to cite Chrys Ingraham's work, it should also consider Elizabeth Freeman's book, The Wedding Complex.

 Last, although they are not as recent, the following pair of articles offer important looks at the production of media "during the wedding" because the authors are not only scholars, they are also practitioners:

Resolutions : contemporary video practices : Free Download, Borrow, and Streaming : Internet Archive

 Thus, these also consider the gaze, the gazes and the composition of the photographs and wedding videos; that is, the kernel of the "mediatisation." Moreover, Ouellette's materialist perspective on the photographs considers the gaze not only from Mulvey's perspective, but also in terms of Berger's earlier work on "publicity" in his germinal *Ways of Seeing*. 

 Where the current paper contributes the most is in terms of the construction of memories, although again, Ouellette's consideration of the "indexical record" is a semiotically grounded study of that very topic. Thus, the paper needs to reconsider its intervention and locate itself among and/or beside the existing literature because there is a conversation to be had, if the author(s) avail themselves of it.

If applicable, is the statistical analysis and its interpretation appropriate?

Not applicable

Is the study design appropriate and is the work technically sound?

Partly

Is the work clearly and accurately presented and does it engage with the current literature?

Partly

Are the conclusions drawn adequately supported by the results?

Yes

Are sufficient details of methods and analysis provided to allow replication by others?

Yes

Are all the source data and materials underlying the results available?

Yes

Reviewer Expertise:

Cultural and Gender Studies, with specialization in contemporary popular culture.

I confirm that I have read this submission and believe that I have an appropriate level of expertise to confirm that it is of an acceptable scientific standard, however I have significant reservations, as outlined above.

## References

[1] : Remaking the White Wedding? Same-Sex Wedding Photographs’ Challenge to Symbolic Heteronormativity. *Gender & Society* .2012;26(6) : 10.1177/0891243212449902 874-899 10.1177/0891243212449902

[2] : Editor’s Pick. *Sociological Perspectives* .2016;59(4) : 10.1177/0731121415601269 721-742 10.1177/0731121415601269

[3] : Ritualized Transmission of Social Norms Through Wedding Photography. *Communication Theory* .2006;16(1) : 10.1111/j.1468-2885.2006.00004.x 31-46 10.1111/j.1468-2885.2006.00004.x

# Response

**Published:** 2024-11-22

**Authors:** Marie-Therese Mäder

**Affiliations:** Philosophy, Ludwig Maximilians University Munich, Munich, Bavaria, Germany

I appreciate your justified and further-reaching critique. The state of research has been completely revised by including relevant literature about wedding photography as you suggested. Specifically helpful is Michele Strano’s concept of descriptive and injunctive norms and values, Katrina Kimport’s observations about the dominance of heteronormativity. Finally, your contribution “Saying ‚I Do‘ All over Again” was incredibly insightful specifically the deliberations of the staged photo session and the material aspects of the wedding as a signifying chain. The indexicality of the wedding photos was also included. The literature review focusses on how norms and values are expressed in wedding photography, and in which sense the photos and videos influence memory processes. It is structured into the following four areas: (1) norms and values, (2) memories, (3) the ritual dimension, and (4) emotions. The construction of memories has been extended by Pierre Nora's concept of "lieux de mémoire". The memory spaces of wedding photography are conceived as cultural archives that contain an individual and a collective dimension. At the same time, the spaces represent different moments in which memory is created. Accordingly, the analysis of the memory process is systematised into the four spaces of production, representation, reception and distribution (the latter two have been grouped together in the analysis because of their overlapping characteristics), which include certain practices. Thanks again for your constructive feedback that has advanced the research. The observations from the interviews were better linked to the theoretical framework. The review deepened and differentiated the results of the field research fundamentally. The results could therefore be better presented and categorised.

# Reviewer report: Reviewer response for version 1

**DOI:** 10.21956/openreseurope.17835.r37094

**Published:** 2024-03-14

**Authors:** Tal Morse

**Affiliations:** 1 Visual culture, Sami Shamoon College of Engineering (Ringgold ID: 42740), Be'er Sheva, South District, Israel; 2 Department of Photographic Communications,, Hadassah Academic College, Jerusalem, Jerusalem District, Israel

This is a very well-written paper that explores the use of photography as a means for constructing and arguably consolidating memories from weddings. It draws on vast and diverse ethnographic research, namely interviews with couples from various backgrounds and their experience of photographing their weddings and utilizing photography to perpetuate these rites of passage. I particularly enjoyed the analysis of the interviews according to the phases of production and reception,

 Given the commercialized aspects of the weddings industry, it might be useful to explore Eva Ilouz’s work on the commodification of romance and other aspects of capitalism in the production and consumption of romantic relations.

 As for mediatization, which seems like a central theoretical framework, I would be helpful to further explain which theory of mediatization is in use. Scholars that have engaged with theories of mediatization have discerned at least three perspectives on this term: *an institutionalist* one, *a social-constructivist* (or cultural) one, and *a material/technological* one. The literature review needs to acknowledge these various perspectives and explain which of them was found fit to this study, and why.

 Lastly, the literature review discusses studies of representation of weddings, but I think it might be a helpful to discuss ethnographic studies of actual weddings, rather than their representation in popular culture.

 As for the term memory spaces that guides the analysis and discussion, I think the article must acknowledge the work of Pierre Nora on memory spaces (Lieu de mémoire) and it needs to explain where and how it diverts from it. On this note, the discussion of the findings needs to delve more into the practice of returning to these memory spaces. The article discusses this practice, but it was unclear to me when and how often do these couple go back to the album and re-consume them (and whether this has changed over time). If, as appears on page 14, some couples hardly do so, it is worth reflecting whether memory spaces is the appropriate concept.

 I would also like to encourage the authors to better clarify and explicate the theoretical contribution of the study and how it helps us to better understand the role of photography in rites of passage and the mediatization of rituals.

 Besides these, there are minor points for the author to consider:

- On page 12, the author writes: “The photo of the kissing newlywed couple exists in almost every wedding photo collection as if the kiss after the wedding has a different quality to the ones that took place before the wedding”. However, since these are photos of rites of passage, there is a significant difference in the social status of the newlywed couple. Before the ceremony, they were not recognized as a couple, and how they are. In some religions, this also affects the legitimacy of the kiss before the ceremony.
- I would consider changing “Results” to “Findings”.

I invite the author to consider the above suggestions to bolster the theoretical contribution of this study. I believe that reflecting on these suggested points will help making a substantial contribution to the study of photography, rituals and mediatization.

If applicable, is the statistical analysis and its interpretation appropriate?

Not applicable

Is the study design appropriate and is the work technically sound?

Yes

Is the work clearly and accurately presented and does it engage with the current literature?

Partly

Are the conclusions drawn adequately supported by the results?

Yes

Are sufficient details of methods and analysis provided to allow replication by others?

Yes

Are all the source data and materials underlying the results available?

Yes

Reviewer Expertise:

Visual culture, mediation of suffering, media and death.

I confirm that I have read this submission and believe that I have an appropriate level of expertise to confirm that it is of an acceptable scientific standard, however I have significant reservations, as outlined above.

## References

[1] : Snapshot Versions of Life. *PopularPress* .1987;

[2] : Going to bed with a camera. *International Journal of Cultural Studies* .2010;13(6) : 10.1177/1367877910376581 637-656 10.1177/1367877910376581

[3] : Digital photography: communication, identity, memory. *Visual Communication* .2008;7(1) : 10.1177/1470357207084865 57-76 10.1177/1470357207084865

[4] : ‘A powerful, spiritual, win-win situation’: commercial authenticity in professional birth photography. *Media, Culture & Society* .2022;44(5) : 10.1177/01634437211060237 951-966 10.1177/01634437211060237

# Response

**Published:** 2024-11-22

**Authors:** Marie-Therese Mäder

**Affiliations:** Philosophy, Ludwig Maximilians University Munich, Munich, Bavaria, Germany

First of all, I appreciate the thoughtful review and the reference to van Dijck J: Digital photography: communication, identity, memory. Visual Communication. 2008; 7 (1): 57-76. Van Dijck's reflections on memory and identification make an important point about the relationship between photography, memory and identity. The completely revised literature review focuses on ethnographic studies of actual weddings, how wedding photography expresses norms and values, and how the photos and videos influence memory processes. The overview of the literature discussed is restructured into the following four areas: (1) norms and values, (2) memories, (3) the ritual dimension, and (4) emotions. The difference between the terms 'mediation' and 'mediatisation' has been defined more clearly and the theoretical concept of mediatisation relevant to the study has been explained in detail with reference to the relevant literature (Knut Lundby 2023; Mark Deuze 2022; Friedrich Krotz 2017; Nick Couldry and Andreas Hepp 2013; Katarzyna Kopecka-Piech 2022). Both approaches to mediatisation, a cultural (social constructionist) and an institutionalist one, are relevant, while the processuality of both is crucial. Both capture the whole process of how media are used to structure, communicate and remember an event. Pierre Nora's concept of "lieux de mémoire" has also been included in the argument. The memory spaces of wedding photography are conceived as cultural archives that contain an individual and a collective dimension. At the same time, the spaces represent different moments in which memory is created. Accordingly, the analysis of the memory process is systematised into the four spaces of production, representation, reception and distribution (the latter two have been grouped together in the analysis because of their overlapping characteristics), which include certain practices. After each section, the main interim results are summarised. How often the couples look at the albums and the role the current research plays on these memory processes is now reflected in the section of the space of reception. (Some couples look at the album regularly or at least once a year. The closer the wedding date is the more often they look at the photos.) The results are synthesized in the conclusion that has been completely revised. The aim was to create cross-connections between the memory spaces of the analysis and to better understand the role of photography in rites of passage and the mediatization of rituals. The thematic areas in the conclusion cover the following topics: (1) The memory of the photo session with the photographer during the wedding day; (2) the photos of the ceremony and their reception; (3) how the media enlarge the wedding experience; (4) the homogenization of memory (and standardisation of emotions); (5) gender differences in the memory space of reception. Two other changes you suggested have been implemented:

- The results section is now changed to “findings”.
- The analysis of the kissing newlywed couple is more elaborated with a references to the rite of passage and the change in social stauts.

Thank you again for your constructive comments. They have really helped to take the research to the next level. I have been able to link the observations from the interviews more closely to the theoretical framework, which has been great. The review has also helped to differentiate the results of the field research in a much more meaningful way. As a result, I have been able to present and categorise the results in a much clearer and more effective way.

# Reviewer report: Reviewer response for version 1

**DOI:** 10.21956/openreseurope.17835.r37818

**Published:** 2024-02-20

**Authors:** Ilya Parkins

**Affiliations:** 1 The University of British Columbia, Vancouver, British Columbia, Canada

This is a very timely set of research data and I think there is a lot of promise in it. In its current form, I don't think all of that potential is borne out. I would like to see a much more robust analysis - there are so many fascinating observations, but they are often not scaled up and analyzed in enough depth. I will elaborate on this below. 

 First, I did want to note that there is a great deal of relevant literature missing from the literature review. The claim that "no scholarly literature has been published so far" about media produced during the wedding is not true. There is a small body of work on wedding photography and videography. For instance, see  [1] published in *Communication Theory*;[2] published in *Women’s Studies in Communication*; Charles Lewis, “Working the Ritual: Professional Wedding Photography and the American Middle Class, [3] in *Journal of Communication Inquiry*; [4]; **[5] in *Visual Resources*; [6] in *Journal of Visual Literacy*; and Matthew Wade and Michael James Walsh, “Their Time and their Story: Inscribing Belonging through Life Narratives and Role Expectations in Wedding Videography.” I think that framing your contribution in dialogue with the findings of these authors is crucial.

 I also think there are some missing references in the work on wedding media, such as Erika Engstrom’s book, *The Bride Factory*, and [7].

 Moving away from the literature review, I really like your explanation of the ways that looking at wedding photography is both about personal/individual memories, and the formation of cultural memory. I do suggest you perhaps choose another way to describe this rather than two-dimensional – the reason is that this term, two-dimensional, is often used to describe photography, so I thought you were referring to the technical qualities of photography.

 On this question of the formation of cultural memory through the wedding photos, though, you make such an interesting observation about the entwinement of these elements, when you introduce the work on mediatization and memory – but then this completely drops out of the analysis. I’m not at all clear how your data is linked to the production of cultural memory. I think this is key – if you show how these seemingly personal looks at wedding photos actually contribute to cultural memory, I think you will make an important contribution. As it is, this is very unclear to me.

 In the remainder of the article, I wanted to see more indications of the significance of what you were describing…for instance, you note that couples were essentially performing for the photographer at certain points during the ceremony, and this seems very notable. What does it mean? How does this contribute to the analysis you are building? As well, the disjuncture between one woman’s description of her own memories of the wedding as just fun and funny, and her wedding video’s focus on cheesy romance, seems really important. To me, this seems like it could be read as exemplary of a subject’s agency in relation to an overwhelmingly ideologically saturated occasion.

 All in all, I wanted to read much more about what it all means. The conclusion could be the place to do this, but I think it would probably either be best done via a) integrating your analysis of the interviews and the data throughout, or b) having a lengthy findings section, in which you really develop your analysis and indicate for the reader why they should be interested in this data. As I say, there’s so much richness here, and I would like the author to really dig in theoretically and make sense of it, showing us how it connects to, contributes to, or broadens our understandings of wedding photography, memory, etc.

If applicable, is the statistical analysis and its interpretation appropriate?

Not applicable

Is the study design appropriate and is the work technically sound?

Yes

Is the work clearly and accurately presented and does it engage with the current literature?

Partly

Are the conclusions drawn adequately supported by the results?

Partly

Are sufficient details of methods and analysis provided to allow replication by others?

Not applicable

Are all the source data and materials underlying the results available?

Yes

Reviewer Expertise:

gender studies, fashion, weddings

I confirm that I have read this submission and believe that I have an appropriate level of expertise to confirm that it is of an acceptable scientific standard, however I have significant reservations, as outlined above.

## References

[1] : Ritualized Transmission of Social Norms Through Wedding Photography. *Communication Theory* .2006;16(1) : 10.1111/j.1468-2885.2006.00004.x 31-46 10.1111/j.1468-2885.2006.00004.x

[2] : Hegemony in the Ideal: Wedding Photography, Consumerism, and Patriarchy. *Women's Studies in Communication* .1997;20(2) : 10.1080/07491409.1997.10162409 167-188 10.1080/07491409.1997.10162409

[3] : Working the Ritual: Professional Wedding Photography and the American Middle Class. *Journal of Communication Inquiry* .1998;22(1) : 10.1177/0196859998022001006 72-92 10.1177/0196859998022001006

[4] : Fashioning Cosmopolitan Citizenship: Transnational Gazes and the Production of Romance in Asian/American Bridal Photography. *Journal of Asian American Studies* .2014;17(2) : 10.1353/jaas.2014.0017 133-160 10.1353/jaas.2014.0017

[5] : Wedding Photography: ‘A Shining Language’. *Visual Resources* .2002;18(1) : 10.1080/01973760290026477 1-16 10.1080/01973760290026477

[6] : Manufacturing the Norm: The Origin and Development of Candid Wedding Photography. *Journal of Visual Literacy* .1998;18(1) : 10.1080/23796529.1998.11674525 15-46 10.1080/23796529.1998.11674525

[7] : Reading Bridal Magazines from a Critical Discursive Perspective.2014; 10.1057/9781137333582 10.1057/9781137333582

# Response

**Published:** 2024-11-22

**Authors:** Marie-Therese Mäder

**Affiliations:** Philosophy, Ludwig Maximilians University Munich, Munich, Bavaria, Germany

First of all I appreciate the thoughtful review that helped me to elaborate the analysis in more depth. First I included all the literature you suggested. Specifically Strano, Bezner, Lieu and Lewis were most insightful for the context of the study’s findings. The literature review has been adapted accordingly and revised completely. The two-dimensional description of personal/individual memories and the formation of cultural memory is now more differentiated. Pierre Nora's notion of cultural archives is included in the discussion and informs the notion of memory considered in the four spaces (production, representation, reception and distribution) of wedding photography. These spaces are cultural archives where the individual and collective dimensions of memory interact. The link between mediatisation and cultural memory, as well as individual memory, is not only sharpened in the theoretical section, but also elaborated in the analysis. It is argued that the cultural practice of adhering to photographic stylistic conventions smooths out the cultural-religious differences between institutionalised ceremonies over time at the macro level. Photographs therefore enrich and connect the cultural memory of weddings regardless of their religious or secular context. As you suggested, the analysis has been further developed by providing more in-depth analysis, structuring and summarising the findings at the end of each section. The conclusion section has been expanded to include a cross-reading of the analysis results. Thank you again for your constructive comments. Your feedback has significantly enhanced the research. The observations from the interviews were more closely aligned with the theoretical framework. Your review allowed for a more nuanced and differentiated analysis of the field research results. The results are now more effectively presented and categorised.
